# A Survey of Traditional Animal-Origin Foods and Preliminary Assessment of Market Characteristics at the Hornbill Festival in Nagaland, India

**DOI:** 10.3390/foods15172991

**Published:** 2026-08-26

**Authors:** Ramesh Vikram, Jyoti Jawla, Yallappa M. Somagond, Laishram Sunitibala Devi, Vangimalu Narsimhappa Narendra, Suchismita Pal, Jagdish Choudhary, Kobu Khate, Keshab Barman, Perumal Ponraj, Patil Shivanagowda Girish

**Affiliations:** 1 Indian Council of Agricultural Research-National Research Centre on Mithun, Medziphema 797106, India; vikki955@gmail.com (R.V.); somagondyallappa@gmail.com (Y.M.S.); thoibi.suniti@gmail.com (L.S.D.); narendravnga@gmail.com (V.N.N.); palsuchismitha98.sp@gmail.com (S.P.); jagdishsyag111@gmail.com (J.C.); kobukhate@gmail.com (K.K.); 2 Indian Council of Agricultural Research, Krishi Bhawan, New Delhi 110001, India; jawlajyoti@gmail.com (J.J.); keshab.barman@icar.gov.in (K.B.); 3 Division of Animal Science, Indian Council of Agricultural Research-Central Island Agricultural Research Institute, Port Blair 744101, India; perumalponraj@gmail.com

**Keywords:** traditional foods, hornbill festival, animal-origin foods, market opportunities, Nagaland

## Abstract

This study documented traditional animal-origin food products and evaluated their market potential at the Hornbill Festival in Kohima, Nagaland, India. Structured face-to-face surveys were conducted over three consecutive festival editions (2023–2025) to record traditional foods, marketing practices, packaging materials, consumer preferences, and pricing. Most meat products were prepared using traditional methods, including hot smoking, drying, and fermentation. Sixteen traditional meat dishes were documented in morungs (traditional Naga huts), alongside several unconventional animal-origin foods. Among 100 surveyed shops each year, pork was the most frequently sold meat (95.4% of shops), followed by beef (46.0%), chicken (44.82%), snails (43.67%), worms (28.73%), and other unconventional animal-origin foods. Pork also accounted for the highest proportion of animal-origin foods sold (36.49%), followed by chicken (17.77%) and beef (16.82%), whereas duck (0.47%) and squirrel (0.23%) represented the smallest shares. Areca nut plates (24.12%) were the most common serving material, followed by aluminum foil containers (22.72%) and banana leaves (20.45%). Mithun, chevon, bird, hornet (insect), and duck dishes were the most expensive. These findings provide baseline information on traditional animal-origin foods and highlight opportunities for developing value-added products and promoting entrepreneurship while preserving indigenous culinary heritage.

## 1. Introduction

Hornbill Festival holds a significant cultural and ecological importance and is celebrated across all major tribes of Nagaland State, India. It is named after the Great Hornbill (*Buceros bicornis*), revered for its beauty, grandeur, and alertness, which commands deep respect from the local communities [1]. The Hornbill Festival, held annually at Kohima, Nagaland State, India attracts a large number of international and domestic tourists and provides a platform for showcasing and promoting traditional products, including animal-origin foods, especially from Nagaland State. During this festival, traditional food products are marketed to tourists as a part of the cultural and gastronomic experience. With an increase in awareness of health and wellness, there is an increasing demand among tourists for natural and organic products [2]. Traditional food products, which are prepared using natural ingredients and processes, have a unique taste and flavor that cannot be replicated by mass-produced commercial meat products. This uniqueness can be leveraged to create a niche market, creating local employment that supports the livelihoods of small-scale producers and farmers.

For indigenous tribal peoples like the Nagas, their knowledge is holistic and traditional, be it food, medicines, values, morals, or other cultural practices. These are inextricably attached to their ancestral lands so that their values remain inseparable from their land and resources. Meat is an essential part of their diet, and several traditional meat products are popular in the region. Some of the important traditional meat products of Nagaland are smoked meat, bamboo shoot with pork, pork with axone, Mithun and fish products [3,4]. Apart from meat products, the local food comprises dried vegetables, vegetable-, bamboo- or pulse-based fermented food products, forest leaves, berries and herbs. In addition, traditional knowledge of insects (dragonflies, beetles, grubs of white ants, grasshoppers, locusts, crickets, stink bugs, grubs of all sorts of bees and wasps, bee comb, silkworms and honey), snails and crabs as food is well established, as these foods have nutritional value and play an important role in the diet of different ethnic groups [5,6,7,8]. There are many studies on edible insects, molluscs and amphibians, traditional fermented foods and meat preservation techniques [9,10,11,12]. However, there is a paucity of information on the available range of traditional animal-origin products, consumer behavior, serving or packaging materials used, and prices at the Hornbill Festival in Nagaland that could help in identifying market opportunities for making business decisions. Documenting these aspects is significant because the festival represents a unique interface between indigenous food traditions, cultural heritage and an expanding tourism-based food market. Such information can provide baseline evidence on the diversity and visibility of traditional animal-origin foods, their consumer acceptance, pricing practices and modes of presentation, while also contributing to the recognition and preservation of indigenous food knowledge. Furthermore, understanding consumer preferences and market practices may help local producers, vendors and policymakers identify opportunities for value addition, improved presentation and sustainable commercialization of these traditional products without compromising their cultural identity [9,10]. In this context, a survey of different morungs (traditional Naga huts) and shops at the Hornbill Festival was conducted to document traditional animal-origin food products, consumer behavior, food preferences, serving and packaging materials, and prices of individual dishes of various animal-origin foods. The novelty of the present study lies in systematically documenting, within the specific setting of the Hornbill Festival, the diversity of traditional animal-origin foods together with consumer preferences, serving and packaging materials, and market prices. To the best of our knowledge, no previous study has comprehensively characterized these interconnected aspects of traditional animal-origin food products presented and marketed at the Hornbill Festival. Thus, the study provides a unique baseline dataset linking indigenous food heritage with consumer behavior and festival-based market practices, thereby offering new insights into the cultural, gastronomic and economic significance of traditional animal-origin foods in Nagaland. This survey represents the first documented record of the traditional meat display at the Hornbill Festival, unveiling its esthetic and anthropological significance.

## 2. Materials and Methods

### 2.1. Study Area

**Figure 1:**
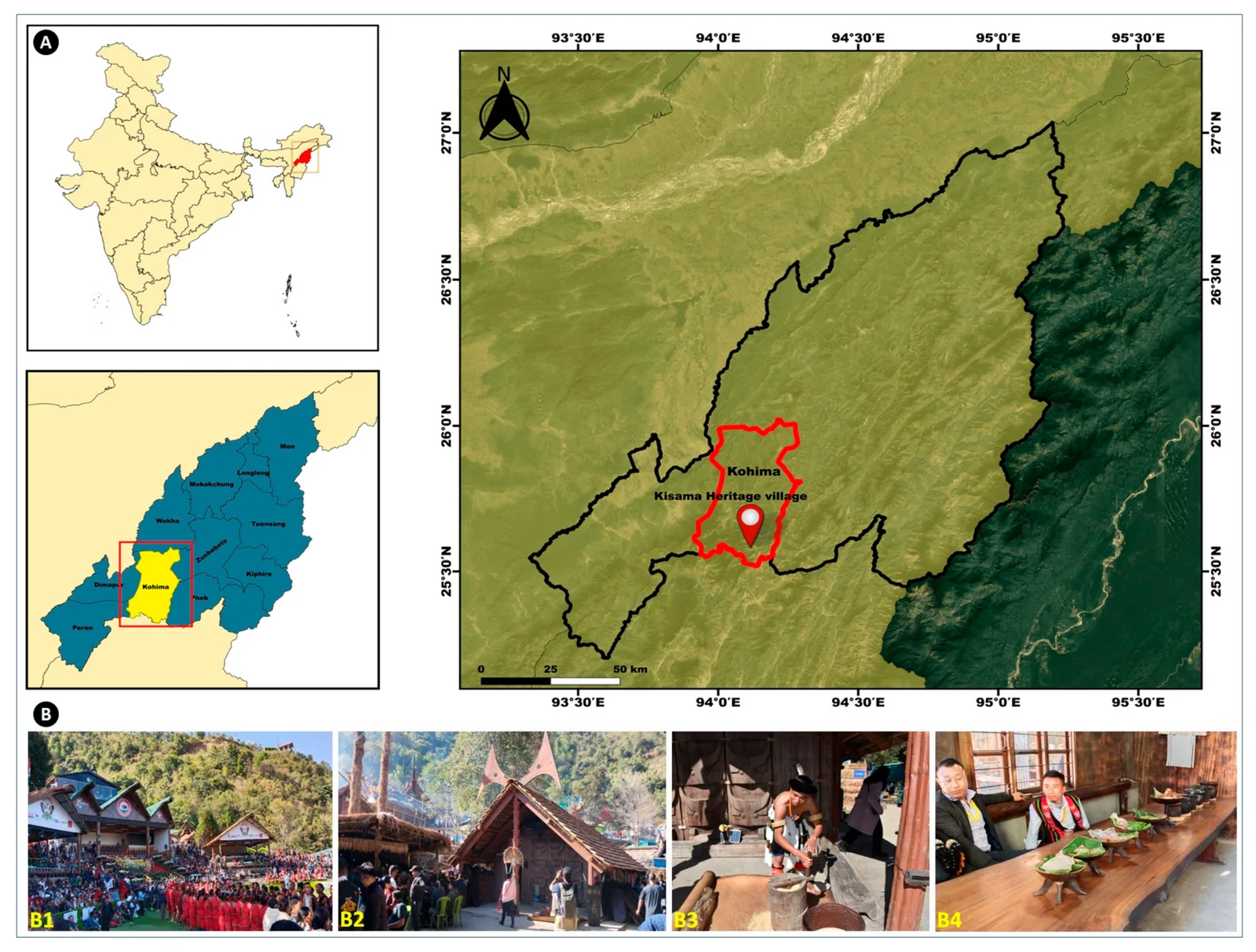
(**A**) Geographical location of Kisama Heritage Village, Kohima district, Nagaland; (**B**) Visual representation of study sites: (**B1**). Hornbill Festival arena; (**B2**). morungs (traditional Naga huts); (**B3**). traditional method of food preparation by ethnic groups; and (**B4**). display of traditional foods in morungs.

Nagaland State is situated in the far northeast of India and has geo-coordinates of 25°10′ N to 27°4′ N latitude and 93°15′ E to 95°20′ E longitude. Covering an area of approximately 1.66 million hectares, the state exhibits altitudinal variations ranging from 100 m to 3826 m above sea level. It is a slender, hilly region running from the northeast to the southwest, extending from the northern continuation of Myanmar’s Arakan Yoma mountain ranges. Nagaland shares borders with Myanmar to the east, Assam to the west and north, Arunachal Pradesh to the north, and Manipur to the south. Nagaland is a biodiversity-rich state with tropical evergreen forests that harbor various medicinal plants. The state comprises sixteen districts: Chümoukedima, Dimapur, Kiphire, Kohima, Longleng, Mokokchung, Mon, Niuland, Noklak, Peren, Phek, Shamator, Tuensang, Tseminyü, Wokha and Zünheboto [13]. Kohima district lies between 25°31′ N–26°2′ N latitude and 93°52″ E–94°17″ E longitude, encompassing a total geographical area of 1317 km^2^ with an elevation of 1444.92 m above sea level. As the capital city, Kohima reflects a cosmopolitan environment, incorporating a mix of all Nagaland tribes. Additionally, it encompasses areas abundant in biodiversity, such as the Dzukou Valley and Mount Japfu. The culinary traditions mirror the rich and varied cultural and ethnic legacy of the area. The survey was conducted in Kisama Heritage Village, Kohima, Nagaland, for three consecutive years, where the Hornbill Festival took place (Figure 1) from 1 to 10 December 2023, 2024 and 2025. Table 1 presents the year-wise visitor profile of the Hornbill Festival during 2023–2025. The visitors went to the morungs to understand the culture and enjoy traditional delicacies.

### 2.2. Naga Ethnic Groups

There are 17 major tribes in Nagaland, namely, Angami, Ao, Chang, Chakhesang, Lotha, Kachari, Konyak, Khiamniungan, Kuki, Pochury, Phom, Sangtam, Rengma, Sumi, Yimchunger, Zeliang and Tikhir, each having unique characteristics with a distinct language, custom, attire and food habits. During the Hornbill Festival, all tribes of Nagaland unite and display traditional handicrafts, vegetables, distinct culinary offerings, traditional dances, and musical performances, along with exhibitions of traditional Naga morungs (traditional Naga huts).

### 2.3. Exploration of Conventional and Non-Conventional Animal-Origin Food Products, and Market Prospects

The information on traditional animal-origin foods was documented by visiting all 18 morungs (n = 18) participating in the Hornbill Festival during 2023–2025. These included morungs representing the 17 major Naga tribes, along with a separate morung representing the Dimasa community of Assam. These morungs constitute the designated tribal morungs at the festival and were therefore included through complete enumeration rather than sampling. From different morungs, the method of preparation of individual traditional animal-origin foods was documented through face-to-face interaction with one representative informant from each morung using a structured survey schedule. Since the ethnic groups speak their distinct languages, the names of the local dishes were kept the same, and the methods of preparation were recorded in their languages and translated into English, along with photographs of the recipes.

For the market-related assessment, 100 food shops were selected randomly during each festival year (2023, 2024 and 2025) from the shops operating at the festival venue, and the pooled sample was considered. The shops were not same across the three years because the participating shops and vendors varied between festival editions. One representative respondent from each selected shop was interviewed. The survey aimed to cover the available food shops as broadly as possible and recorded the animal-origin foods offered, sale preference (%), meat share (%), consumer preference (%) (based on the proportion of dishes sold), serving/packaging materials (%), and price variation of food dishes. Thus, the study included 18 morungs and 100 randomly selected shops in each year, with the morungs representing the participating tribal groups and the shops representing the festival food market.

$$\mathrm{Sale}\;\mathrm{prevalence}\;(\%)=\frac{Number\;of\;shops\;selling\;a\;particular\;animal-origin\;food}{Total\;number\;of\;shops\;surveyed}\times 100$$

$$\mathrm{Meat}\;\mathrm{share}\;(\%)=\frac{Quantity\;of\;meat\;from\;particular\;animal\;species}{Total\;quantity\;of\;meat\;from\;all\;animal\;species}\times 100$$

$$\mathrm{Consumer}\;\mathrm{preference}\;(\%)=\frac{Number\;of\;dishes\;sold\;for\;a\;specific\;animal-origin\;food\;category}{Total\;number\;of\;shops\;surveyed}\times 100$$

$$\mathrm{Serving}/\mathrm{packaging}\;\mathrm{material}\;(\%)=\frac{Number\;of\;shops\;using\;particular\;serving/packaging\;material}{Total\;number\;of\;shops\;surveyed}\times 100$$

### 2.4. Statistical Analysis

Statistical analysis was carried out using SPSS version 20, (IBM Corporation, Chicago, IL, USA). Descriptive statistics were calculated for the price of individual meat products from different animals. The results are presented as mean values ± standard error of the mean (SEM).

## 3. Results

### 3.1. Traditional Animal-Origin Food Products Documented at the Hornbill Festival

**Figure 2:**
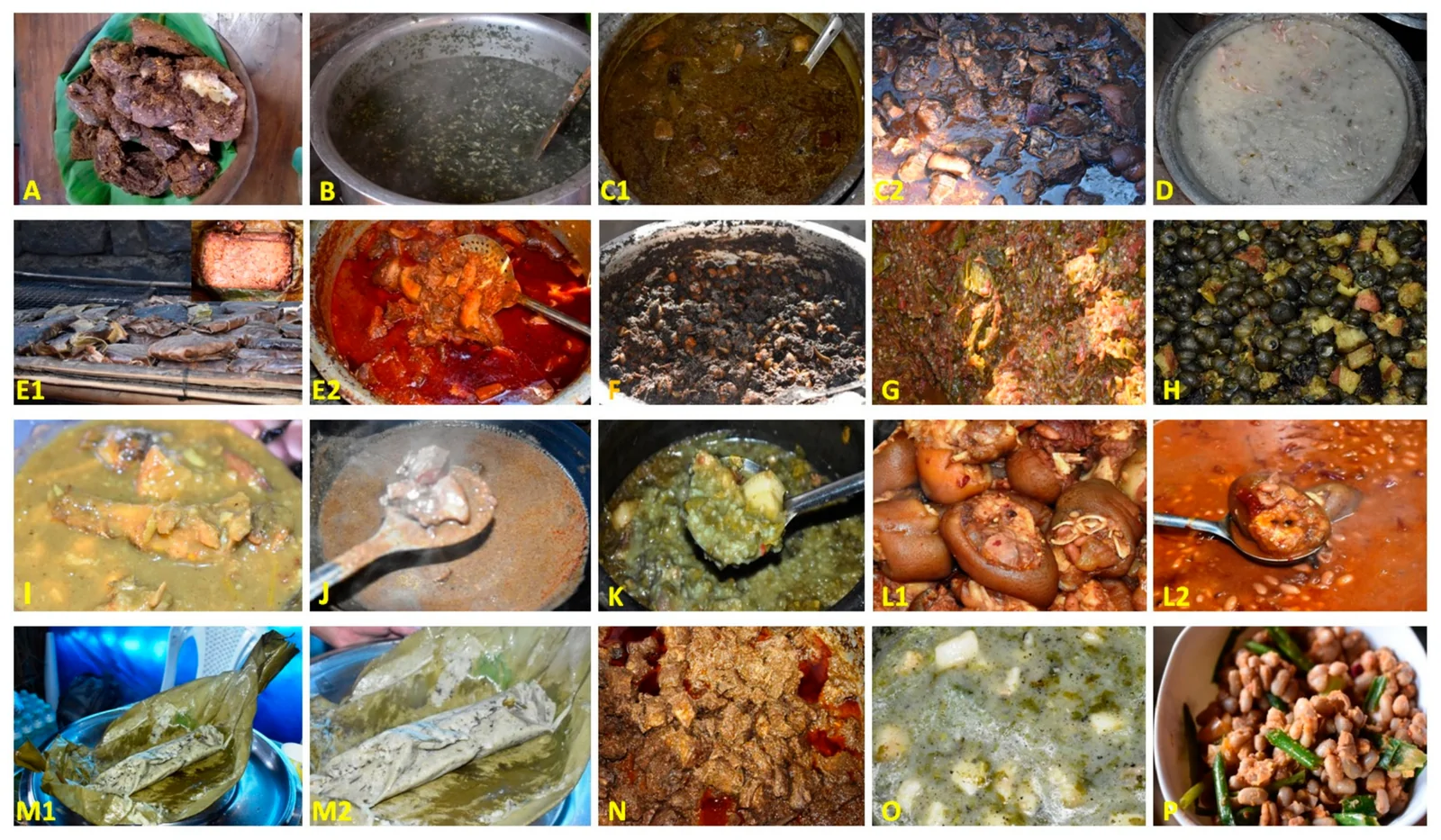
Traditional meat dishes documented in morungs (traditional Naga huts): (**A**) moudi; (**B**) galho; (**C1**,**C2**) anishi/nuoshi; (**D**) amrusu; (**E1**) traditional axone (fermented soybean) preparation involves wrapping boiled soybeans in banana or *Stachyphrynium placentarium* leaves and fermenting them above a fireplace for a week; (**E2**) smoked pork with axone; (**F**) benjung han; (**G**) machi han; (**H**) snail with smoked and dried pork; (**I**) daona hon; (**J**) smoked pork with sesame; (**K**) mepoh; (**L1**,**L2**) pig trotters in kholar beans; (**M1**,**M2**) anphet (aan-phat); (**N**) smoked pork with perilla; (**O**) wak kappa or pork kappa; and (**P**) nasang süru/vin süru.

**Figure 3:**
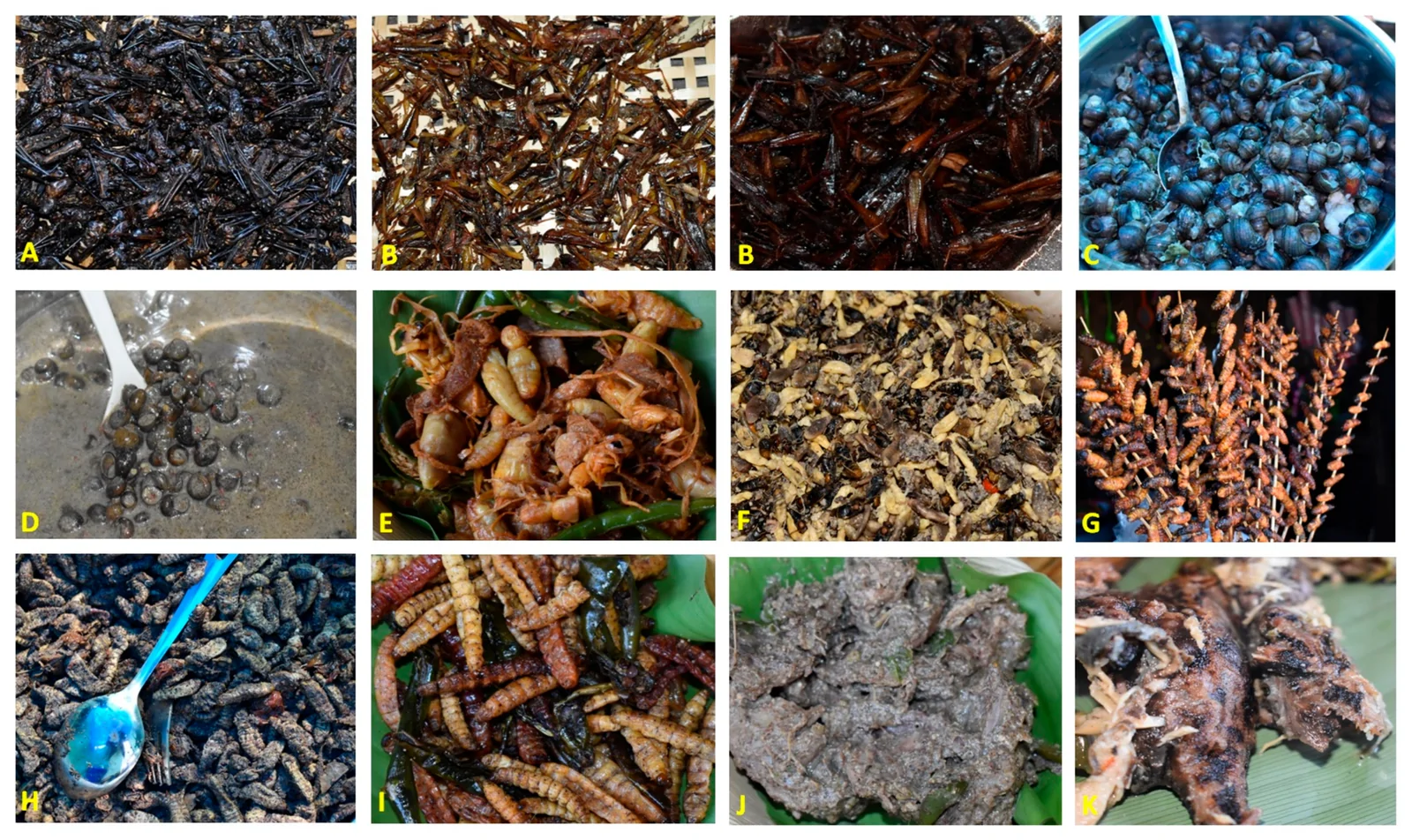
Unconventional animal-origin foods: (**A**) spider; (**B**) grasshopper; (**C**) boiled snail; (**D**) snail curry; (**E**) hornet fry; (**F**) hornet and larva fry; (**G**) fried silkworm; (**H**) boiled silkworm; (**I**) woodworm; (**J**) bird; and (**K**) squirrel.

During the Hornbill Festival surveys in 2023, 2024 and 2025, sixteen types of traditional animal-origin food recipes consumed by different ethnic groups of Nagaland were documented in morungs (traditional Naga huts) (Table 2). The majority of meat products were prepared using traditional methods such as hot smoking (75–80 °C), drying, or fermenting. The traditional meat dishes documented in morungs were moudi, galho, anishi, amrusu, smoked pork with axone, benjung han, machi han, snail with dry pork, daona hon, smoked pork with sesame, mepoh, pork trotters in kholar beans, anphet, perilla with smoked pork, wak kappa and nasang süru (Table 2; Figure 2). In addition, various unconventional animal-origin foods such as worms (silkworms and woodworms), arthropods (spider), insects (grasshoppers and hornets), dogs, squirrels and birds were recorded (Figure 3). Some of the recipes (e.g., moudi and benjung han) had complex methods of preparation with several traditional seasonings. The detailed recipes for the sixteen traditional dishes are described in Table S1.

### 3.2. Sale Preference, Meat Share and Consumer Preference of Animal-Origin Foods

**Figure 4:**
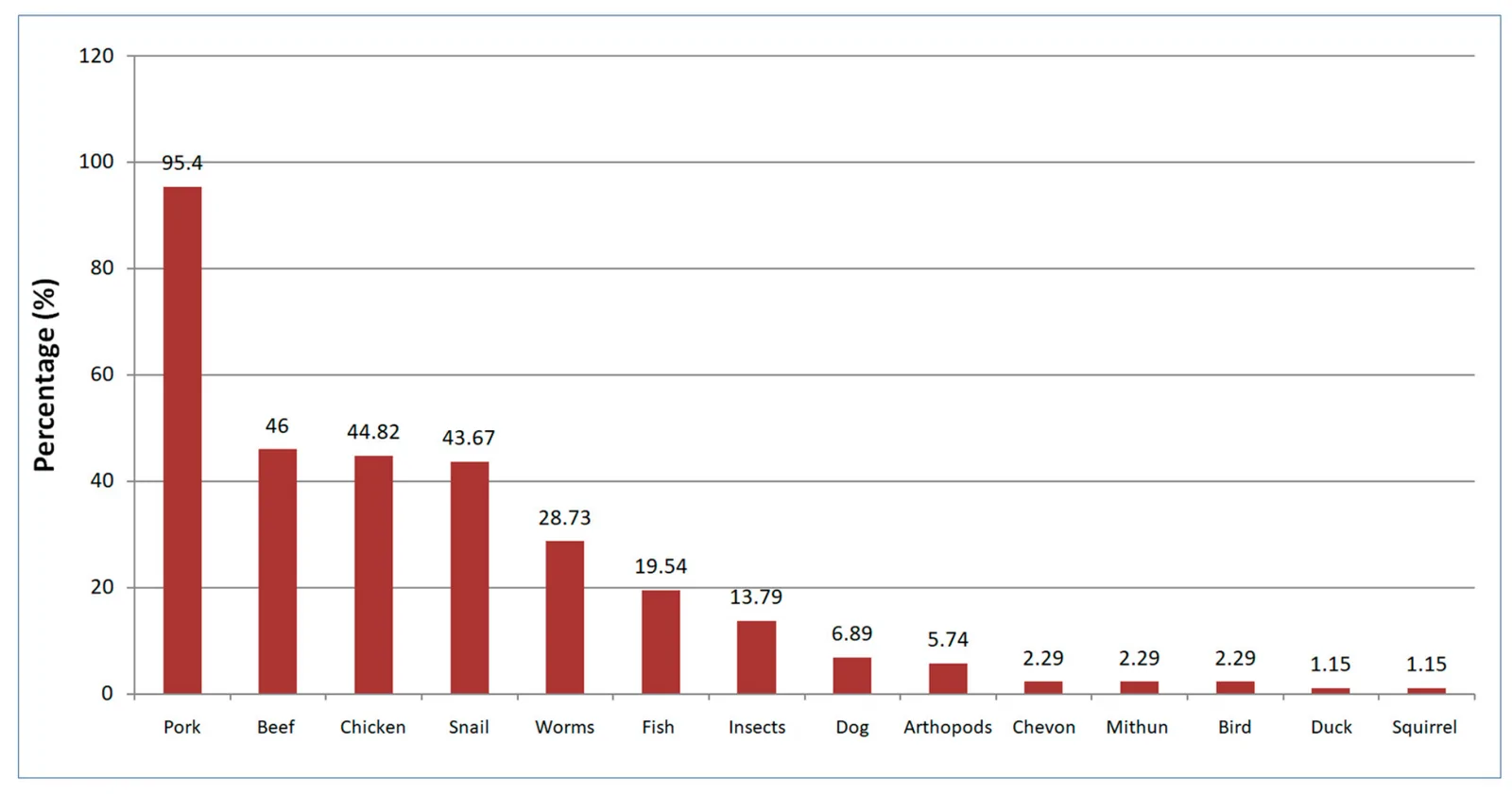
Prevalence of the sale of meat from different animal species among surveyed shops at the Hornbill Festival in 2023, 2024 and 2025, Nagaland.

**Figure 5:**
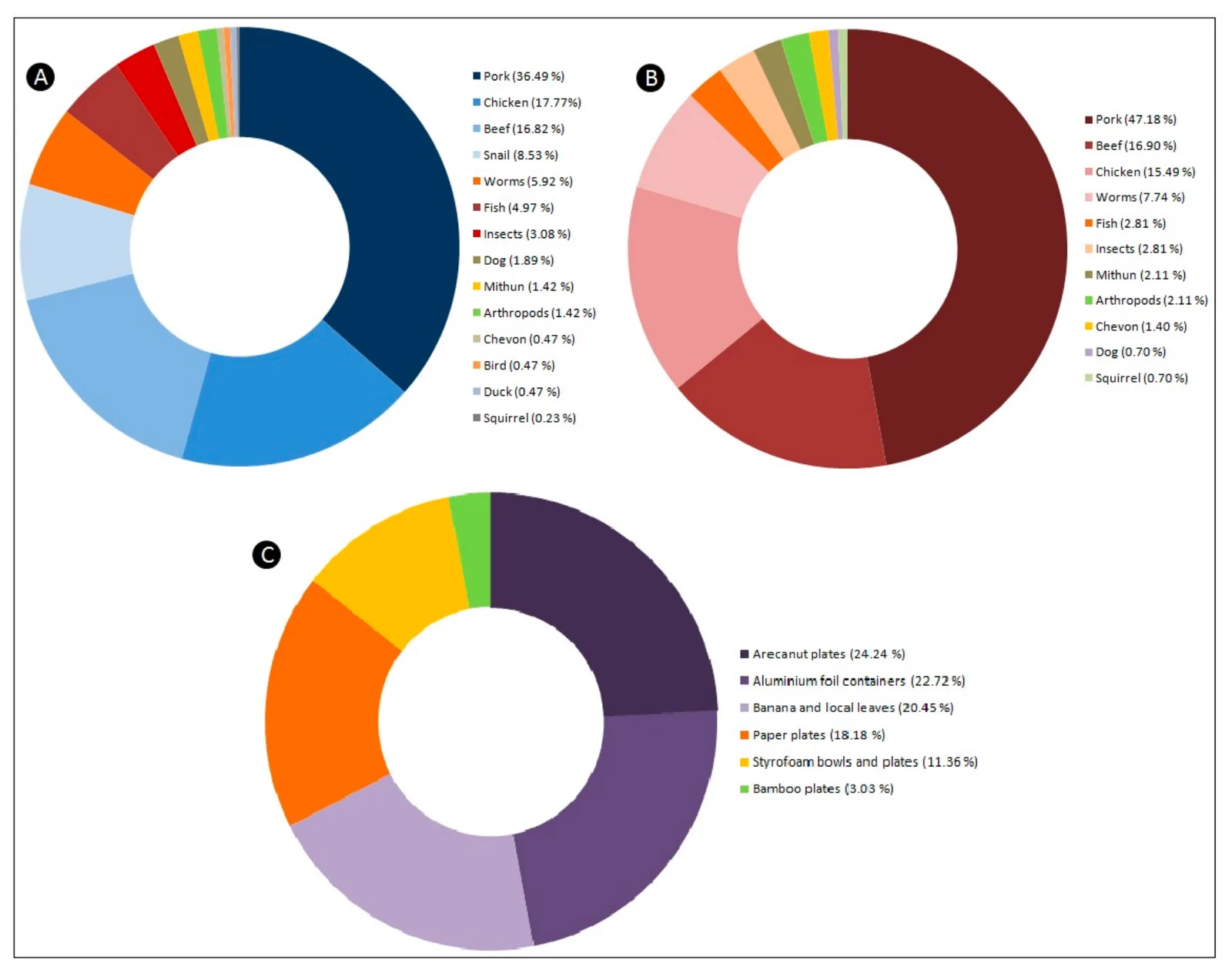
(**A**) Percentage share of meat from different species; (**B**) consumer preference for dishes from different species; and (**C**) percentage of shops using food serving or packaging materials at the Hornbill Festival in 2023, 2024 and 2025, Nagaland.

In accordance with market potential, Figure 4 illustrates the distribution of surveyed stores that offered various types of meat from different animals. Among the surveyed shops, the maximum prevalence of the sale of pork was recorded (95.4% of the shops), followed by beef (46%), chicken (44.82%), snail (43.67%), worms (28.73%) and other unconventional animal-origin foods. Figure 5A illustrates the percentage share of meat from different animals in the festival, with pork leading at 36.49%, followed by chicken (17.77%) and beef (16.82%). Duck and squirrel had the lowest shares at 0.47% and 0.23%, respectively. The consumer preference for pork dishes was highest (47.18%), followed by beef (16.90%), chicken (15.49%) and other unconventional animal-origin foods (Figure 5B).

### 3.3. Serving and Packaging Materials Used for Traditional Foods

Figure 5C depicts the percentage of shops using serving or packaging materials, with areca nut plates having the highest utilization at 24.24% of shops, followed by aluminum foil containers (22.72%) and banana and local leaves (e.g., *Stachyphrynium placentarium*) (20.45%). The use of non-biodegradable Styrofoam bowls and plates were in 11.36% shops.

### 3.4. Price Variation of Animal-Origin Food Dishes

**Figure 6:**
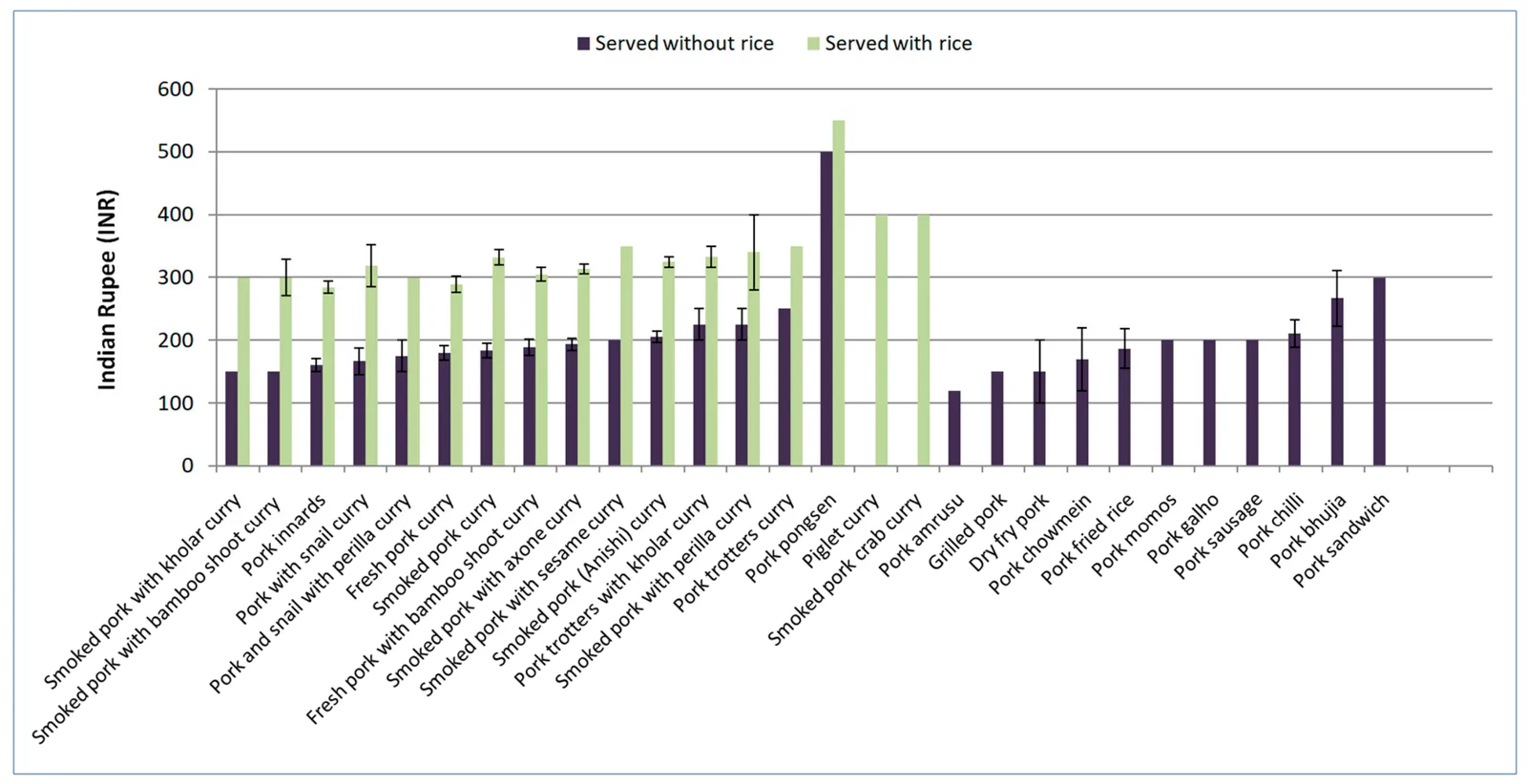
The price of twenty-eight different pork dishes recorded at the Hornbill Festival in 2023, 2024 and 2025, Nagaland (mean ± SEM).

**Figure 7:**
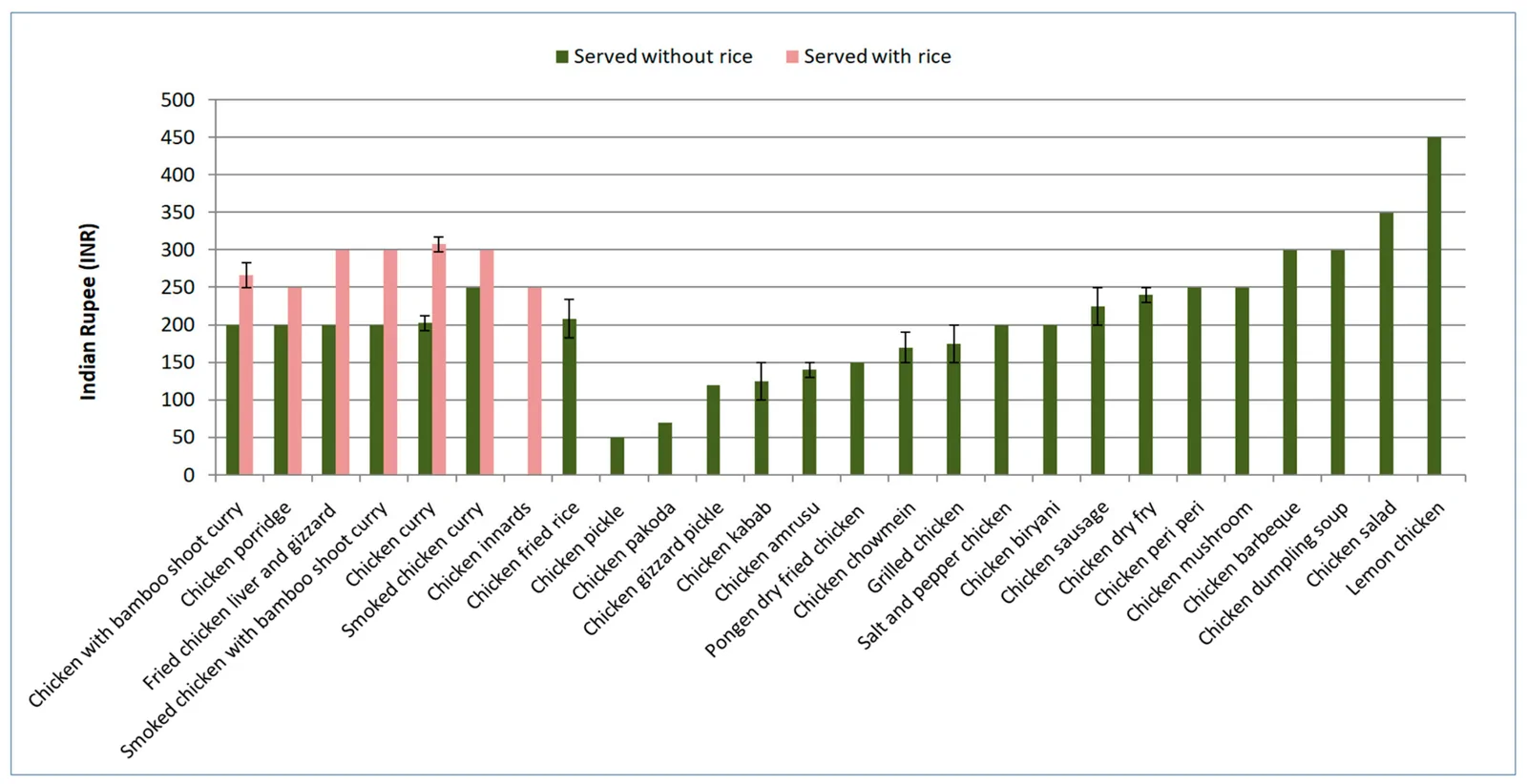
The price of twenty-six chicken dishes recorded at the Hornbill Festival in 2023, 2024 and 2025, Nagaland (mean ± SEM).

**Figure 8:**
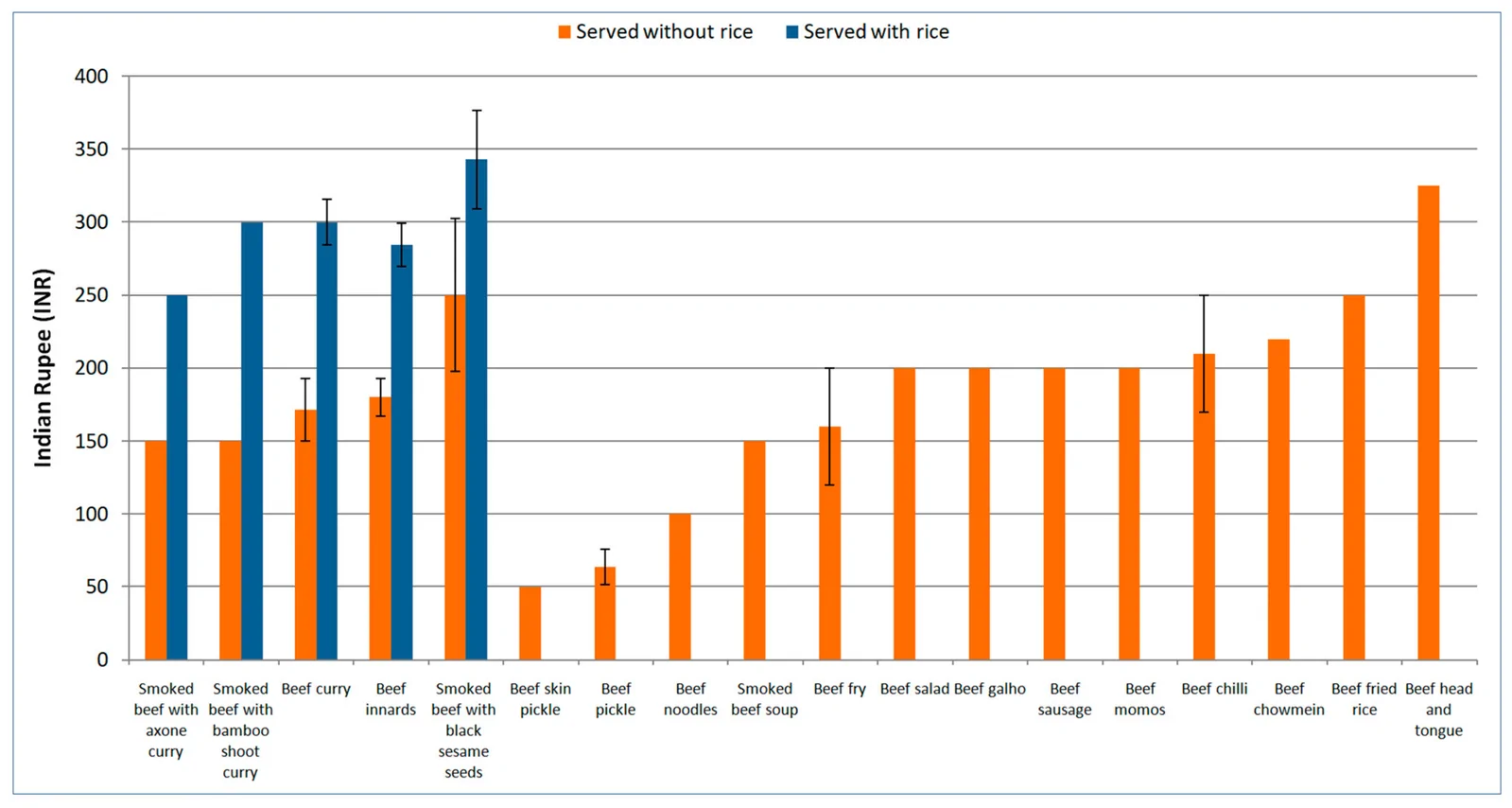
The price of eighteen beef dishes recorded at the Hornbill Festival in 2023, 2024 and 2025, Nagaland (mean ± SEM).

**Figure 9:**
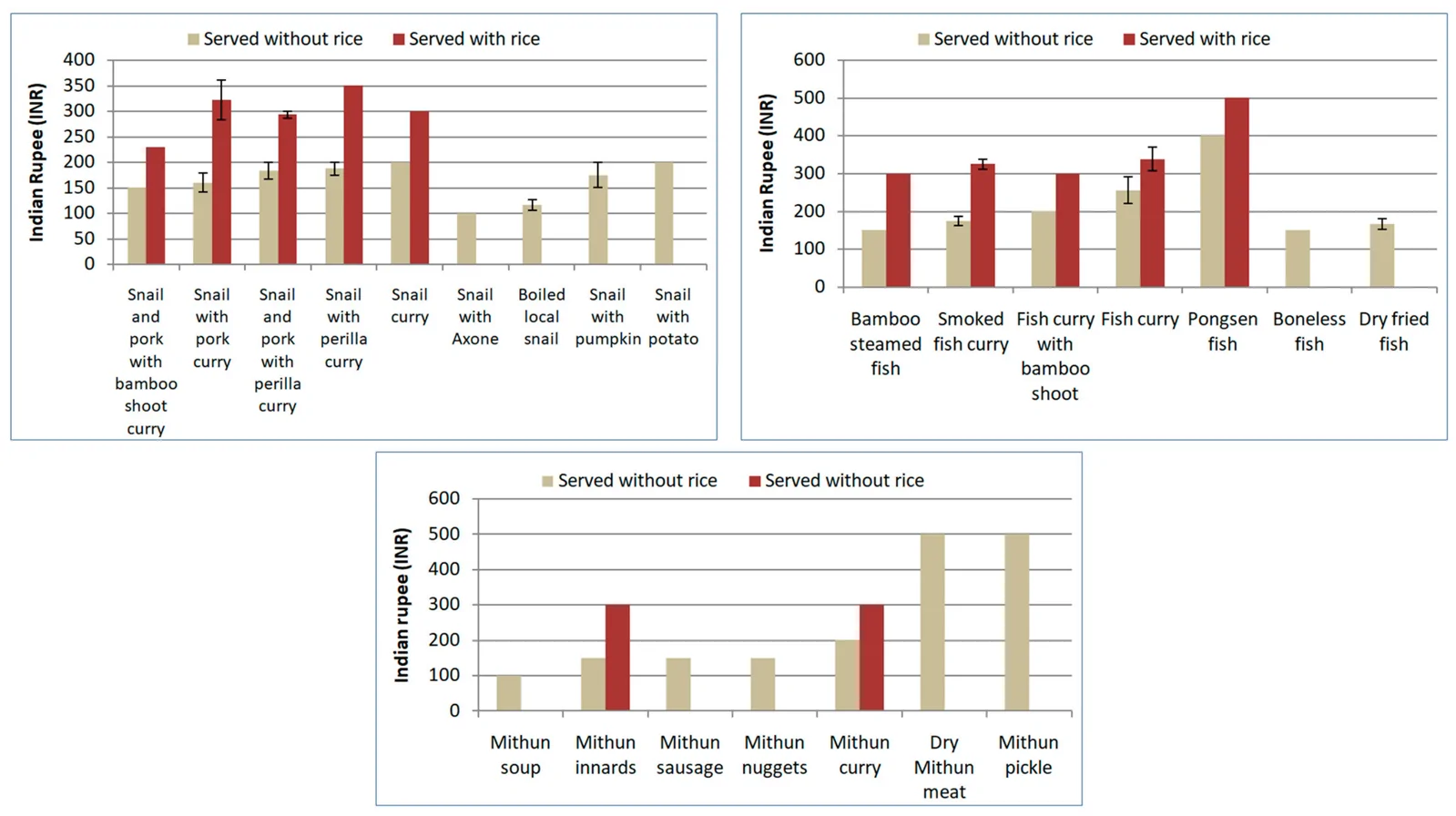
The price of nine snail, seven fish and seven Mithun dishes recorded at the Hornbill Festival in 2023, 2024 and 2025, Nagaland (mean ± SEM).

The mean ± SEM prices of twenty-eight different pork dishes and twenty-six chicken dishes are presented in Figure 6 and Figure 7, respectively. Figure 8 and Figure 9 illustrate the mean ± SEM prices of eighteen beef dishes and nine snail, seven fish and seven Mithun dishes, respectively. Chevon was sold at the highest price [Indian Rupees (INR) 300 to 400], followed by duck (INR 350) and Mithun (INR 300 to 500). The price of individual dishes fluctuated depending on whether they were served with or without rice. The unconventional animal-origin foods included worms (silkworms and woodworms: INR 150 to 200), arthropods (spiders, crabs and prawns: INR 100 to 200), insects (grasshoppers: INR 100 to 200 and hornets: INR 400 to 500), dogs (INR 250 to 350), squirrels (INR 250 to 350) and birds (INR 400 to 500).

## 4. Discussion

This is the first survey that recorded the traditional meat display at the Hornbill Festival for three consecutive years. This festival is attractive as it creates an opportunity to observe the cultural practices of different Naga tribes surrounding the preparation and presentation of meat products. Prominently displayed meats were preserved through age-old techniques like hot smoking and fermentation, techniques passed down through generations (Figure S1). Smoking increases shelf life through the effective broad-spectrum antimicrobial action of phenolic and carbonyl compounds (guaiacol, syringol and catechol) and organic acids (lactic and acetic) generated during combustion [14,15]. These compounds synergistically disrupt bacterial membranes and prevent growth. Furthermore, smoking improves the shelf life of meat by lowering lipid oxidation and water activity [16,17]. Meat preserved through fermentation produces organic acids like lactic acid through enzymatic action, creating an acidic environment that impedes microbial activity and therefore preserves meat [18,19]. The presence of lactic acid bacteria plays a crucial role in this process, contributing to both preservation and the production of nutritious fermented food, and these bacteria have been isolated from indigenous fermented bamboo products [20]. Moreover, smoking imparts flavors to the meat due to the presence of compounds like guaiacol in smoke [21] and the production of peptides and amino acids during fermentation [22,23]. Beyond this, these practices are also used by communities to reserve surpluses for times of scarcity, preserve seasonal foods and facilitate convenient food transport in remote geographical locations of the villages [3]. Importantly, further potential lies in exploring additional benefits other than those acknowledged by the Naga ethnic groups. According to Chai et al. [24], fermentation enhances crucial nutrient bioavailability, generates bioactive peptides with antioxidant and anti-inflammatory effects, and opens avenues for the development of nutraceutical and functional foods. Smoking meat with various spices not only adds distinct color and aroma but also aids in the digestion of essential amino acids [25]. Nevertheless, this practice may also introduce harmful substances such as polycyclic aromatic hydrocarbons or nitrosamines due to the incomplete combustion and pyrolysis processes applied to the wood [26]. Modern smoking methods with separate chambers safeguard against these risks, preserving both safety and flavor [16]. In addition, isolation of pathogenic bacteria like *Salmonella*, *Aeromonas*, *Klebsiella*, *Bacillus cereus* and *Proteus mirabilis* in traditional fermented foods such as soybean (axone), bamboo, fish, and pork indicates a risk of food-borne illness [27,28]. Specific microbial investigations of axone have further highlighted the food safety concerns associated with traditional fermentation. Singh et al. [27] who analyzed 191 axone samples from seven Naga tribes, reported aerobic mesophilic counts of 6.44–12.98 log_10_ CFU/g and staphylococcal counts of 5.69–7.56 log_10_ CFU/g, with *Salmonella*, *Aeromonas* and *Klebsiella* also detected. These findings emphasize the importance of hygienic processing, controlled fermentation and appropriate storage of traditional fermented foods [27]. Although smoking, drying and fermentation were documented as important traditional processing methods in the present survey, specific hygienic conditions, processing temperatures, cold-chain management, handling practices and product exposure during sale were not directly assessed. Therefore, the present study cannot establish the microbiological or chemical safety of these products. Future studies should evaluate these parameters, including good hygienic practices, controlled fermentation and HACCP-based risk assessment, to identify potential contamination risks and develop appropriate food safety interventions while retaining traditional processing practices.

The traditional methods of meat preparation techniques are often passed down through generations and are deeply rooted in Nagaland culture [12]. The traditional meat display showcased a variety of meat products, including pork, beef, chicken and unconventional animal-origin foods. The meat display at the festival provided an opportunity to observe and appreciate the traditional practices and the cultural significance of meat in Naga cuisine. Through this display, the unique cultural heritage of the Naga tribes is celebrated and shared with visitors from around the world, fostering a greater understanding and appreciation of the rich diversity of cultures in India. Sixteen types of traditional animal-origin food recipes consumed by different ethnic groups of Nagaland were documented. Although the traditional recipes were recorded only during the Hornbill Festivals, they can be considered representative of the entire Nagaland for these particular ethnic groups. The local cuisine varied from tribe to tribe, and meat played a significant role in these Naga cuisines. In most prepared cuisines, both vegetables or legumes and meat are usually cooked together. Cooking vegetables or legumes with meat provides a scientifically supported way to enhance flavor, boost nutrient intake, improve digestion, reduce disease risks, manage weight, and have an antioxidant effect [29,30]. In one dish featuring smoked pork, *Perilla frutescens* (L.) seeds, sourced from a herb of the Lamiaceae family, were utilized. Perilla seed oil, rich in polyunsaturated fatty acids, notably α-linolenic acid, demonstrates antioxidant, anti-inflammatory, and antimutagenic properties [31,32]. These seeds can act as novel fat replacers, offering high nutritional value with around 35–45% oil content and enhancing meat quality [33]. The meat (pork, chicken, beef, Mithun, fish, etc.) used in traditional dishes was often smoked and dried or fermented, and different ethnic groups have different cooking procedures, especially regarding the types of seasonings used. Some dishes involving pork were prepared with bas tenga (fermented bamboo shoots), axone (fermented soybean) and anishi (fermented Colocasia leaves) with smoked pork [34]. However, the nutritional composition and quality of these preserved foods are not very well known; therefore, it is high time to understand the microbiology of fermented foods and the nutritional composition of animal-origin foods. The documentation of traditional animal-origin foods is also necessary for cultural preservation, economic opportunities (commercialization) and sustainable agriculture. In addition, culinary knowledge and other traditional knowledge of wild edible plants are passed on from generation to generation orally. The transmission of traditional knowledge regarding the identification of edible plants and food preparation methods may face significant challenges when younger generations show disinterest in acquiring such information [35].

Though various non-conventional animal-origin foods, such as worms, arthropods, and insects, contribute to the dietary composition of the Naga ethnic groups, edible insects remain the most critical dietary component, particularly for rural communities with limited access to conventional sources of animal protein. Preparation methods vary, with most insects cooked, fried, or roasted. However, some, like ant, bee, wasp, and hornet pupae, are traditionally consumed raw [9]. The use of insects as food by Naga ethnic groups goes way back to traditional times. As conventional sources of animal-origin foods are not always available to rural people, communities turn to unconventional options like insects to supplement their diets. Kohler et al., Nongonierma et al., and Zielinska et al. [36,37,38] reported the advantages of insects as a protein source. Compared to traditional livestock, insects offer the dual advantage of high protein content and superior production efficiency, making them a promising and readily available alternative for these communities. In Nagaland, particularly for rural communities with limited economic means, edible insects are still recognized and remain a crucial source of nutrition and income. Various ethnic groups traditionally integrate diverse insects into their diets, including beetles, dragonflies, ant grubs, grasshoppers, locusts, crickets, stink bugs, bee and wasp larvae, and even honey and bee comb [5,6,7,8]. Mozhui et al. [9] documented in detail the traditional knowledge of the utilization of edible insects and worms in Nagaland, highlighting the potential of proper packaging to extend shelf life and elevate edible insects and worms to staple food status. Currently, this insect-based food tradition is undergoing exciting transformations. New techniques, such as those combining protein-processing technologies with insects [36,39], pave the way for the development of innovative food products [40]. Insects can be integrated into familiar dishes while emphasizing their practical value, including their nutritional benefits and environmental sustainability [41,42]. This can stimulate consumer demand and create even greater income generation opportunities within communities. This approach can position Nagaland as one of the leaders in a sustainable and diverse global food system where insects contribute not only to local food security but also to a wider, more sustainable future. Entomophagy is promoted globally to address future food insecurity; however, indiscriminate insect collection poses risks like overharvesting, ecological harm, and the potential consumption of insects contaminated by pesticides or disease-causing agents [43]. Apart from the above, dogs and wild animals are part of the diet of Naga ethnic groups [3,44]. Their use as food highlights the importance of considering conservation, sustainable utilization and veterinary public-health aspects, particularly the potential risks associated with wildlife-derived foods and zoonotic or food-borne infections. These aspects were not directly assessed in the present study and warrant further investigation.

Among the total surveyed shops, pork was sold in 95.4% of them, reflecting its deep cultural and economic significance in Nagaland. Pig rearing and pork consumption are fundamental aspects of life in Nagaland, with pork being preferred over other meats [45]. This dominance is further evident as the % share of pork is the highest, and pigs alone account for 72% of the total livestock population in Nagaland as per the 20th Livestock Census [46]. However, recent years have witnessed a nearly 42% decline in the pig population since 2007 and a nearly 20% decline since 2012. Pork production mirrored this trend, declining from a peak of 60.45 thousand metric tons in 2014–2015 to 15.77 thousand metric tons in 2018–2019 [45]. To address this situation and ensure the sustainability of this critical industry, live pigs are currently being imported from various parts of India to meet the demand. Our study found a similar trend, with pork dishes identified as the most preferred by consumers and pork being sold in the maximum number of shops, followed by beef and poultry [34]. Therefore, it necessitates a multifaceted approach for the future growth and viability of the pig industry. One promising avenue lies in utilizing traditional preservation techniques like smoking and fermentation [47]. These methods not only extend shelf life but also minimize waste and yield a variety of value-added products with a broader market reach and enhanced consumer appeal. Empowering and equipping local entrepreneurs through capacity building in processing, packaging, and marketing can further strengthen the value chain, generate additional livelihoods, and contribute to regional economic growth [34]. Specific industrialization opportunities include the development of value-added products such as smoked and fermented meat products, ready-to-eat traditional meat dishes, vacuum-packed or retort-packed products, meat pickles and standardized spice-based meat products with improved shelf life and packaging. Local entrepreneurship training programs could focus on hygienic processing, standardized recipes, food safety and quality control, packaging, labeling, branding, cost management and digital marketing. Such training could be implemented through collaboration among ICAR institutes, veterinary and food technology institutions, local self-help groups and tribal entrepreneurs. These interventions could help transform traditional food knowledge into commercially viable products while maintaining the cultural identity and authenticity of Naga traditional foods. These initiatives not only attract a wider consumer base but also mirror contemporary market trends [45]. Additionally, exploring bioactive peptides produced through fermentation offers significant potential for the development of functional food products with added health benefits [48]. This response to the increasing consumer demand for products enriched with functional properties presents an exciting opportunity for the industry.

Interestingly, Mithun (*Bos frontalis*), the state animal of Nagaland and Arunachal Pradesh, plays a significant role in local cultural cuisine. Mithun meat contains a higher protein content (14–19%), lower fat levels (0.4–3.58%), and a lower carbohydrate content (0.06–4.97%) compared to other meats [49,50,51]. Further, recent nutritional profiling of Mithun meat demonstrated that lysine and leucine were the predominant essential amino acids, while glutamic acid and aspartic acid were the major non-essential amino acids. The study also characterized the fatty acid profile of Mithun meat, further supporting its nutritional value as a traditional animal-origin food [52]. It also offers a higher vitamin content, with thiamine (vitamin B1) ranging from 0.46 to 0.75 mg/100 g, riboflavin (vitamin B2) from 1.17 to 1.88 mg/100 g, and niacin (vitamin B3) from 3.44 to 4.11 mg/100 g [53,54]. Despite these advantages, Mithun meat and meat products were found in only 2.29% of surveyed shops (ICAR-NRC on Mithun & another entrepreneur). The probable reason for this is that Mithun is considered a valuable asset in Naga culture and is often kept as a status symbol or for ceremonial purposes. Therefore, it is not as readily available for consumption as other types of meat, such as pork, beef or chicken, which are more commonly consumed in Nagaland. Recently, the Food Safety and Standards Authority of India (FSSAI) approved Mithun as a food animal vide gazette notification number CG-DL-E-23022023-243823 dated 22 February 2023 [55]. This decision, coupled with the positive consumer response observed in earlier ICAR-National Research Centre on Mithun stalls, underscores the potential for Mithun meat to contribute significantly to the local economy through sales and exports. Therefore, unlocking the full potential of Mithun meat necessitates the strategic development and subsequent promotion of value-added Mithun products. By expanding their culinary appeal and diversifying market reach, these products can not only significantly enhance the livelihoods of local communities but also contribute to the preservation of this cherished cultural and culinary heritage.

Among the food serving materials (Figure S2), biodegradable areca nut plates contributed to 24.24% of their use in serving meat products. Banana and local leaves were used in up to 20.45% of cases, and the use of plant leaves as dining plates and food wraps is a traditional practice in northeast India. The long-standing tradition has its own cultural, religious, medicinal, and socioeconomic importance in India [56]. In comparison with plastic plates, leaf plates and cups offer many advantages, such as renewability, biodegradability, non-toxicity and antioxidant abundance, in addition to their religious, medicinal, and socioeconomic importance in Indian culture [56]. From an environmental perspective, biodegradable areca nut and leaf-based tableware offers advantages over non-biodegradable expanded polystyrene foam, as these plant-based materials are renewable, biodegradable and can decompose naturally after disposal. In contrast, expanded polystyrene is persistent in the environment, is difficult to degrade and may contribute to long-term solid waste accumulation. Therefore, the greater use of biodegradable leaf-based serving materials observed during the Hornbill Festival may provide a more environmentally sustainable alternative to foam-based tableware, particularly at large-scale cultural and tourism events [56]. The use of non-biodegradable Styrofoam bowls and plates was proportionately lower at the festival. Plastic was only used as a packaging material for ready-to-eat meat products, meat pickles or meat chutneys. Pork, beef and chicken were sold at an affordable price compared to other dishes. Earlier studies also indicated that pork was sold at a higher price compared to beef or broiler chicken; however, it was lower in price than local chicken, dog meat, chevon or Mithun meat [57]. Due to the lower availability of conventional and unconventional animal-origin foods, there is always a demand, and prices are up to double those prevailing in the other parts of the country. As these were festival days, the recorded prices are expected to exhibit an increase relative to the standard regular selling prices. The elevated expense of unconventional animal-derived foods, such as hornets, squirrels, and birds, is linked to the consistent unavailability of specific food sources.

A limitation of the present study is that the market-related assessment was primarily descriptive and did not constitute a comprehensive market analysis. Detailed evaluation of market drivers, consumer segmentation, willingness to pay, SWOT analysis, competitive positioning, economic feasibility, business potential, and year-wise trends in sales, consumer preferences, prices and packaging practices would require additional primary data and appropriately structured longitudinal sampling. Therefore, the present findings should be considered a preliminary baseline for future comprehensive market and value chain studies on traditional animal-origin foods in Nagaland.

## 5. Conclusions

The traditional meat display at the Hornbill Festival served as an important cultural preservation effort, helping to showcase the traditional practices and knowledge surrounding the preparation and presentation of traditional products, including animal-origin foods in Nagaland. The unique flavors, textures, and cultural significance of traditional meat in Naga cuisine were celebrated, preserving the traditional knowledge and practices for future generations to enjoy and appreciate. The survey conducted at the festival documented sixteen different traditional meat dishes. The sixteen traditional meat dishes documented in morungs (traditional Naga huts) were moudi, galho, anishi, amrusu, smoked pork with axone, benjung han, machi han, snail with dry pork, daona hon, smoked pork with sesame, mepoh, pork trotters in kholar beans, anphet, perilla with smoked pork, wak kappa and nasang süru. In addition, various unconventional animal-origin foods were recorded. Pork was the most prevalent meat sold in the shops along with the highest preference in all years. Traditional methods such as hot smoking and drying or fermenting were commonly used for meat preparation. Areca nut plates and local or banana leaves were the most commonly used traditional meat serving materials. The price of meat dishes varied depending on the type of meat and the method of preparation. Beyond documenting culinary diversity, this study establishes a baseline for safeguarding traditional food knowledge while recognizing the potential of these products to contribute to local livelihoods and culturally grounded food entrepreneurship. Future research integrating food safety, sustainability, consumer behavior and comprehensive market assessment can help translate this documented culinary heritage into responsible and sustainable food sector opportunities.

## Figures and Tables

**Table 1:** Year-wise visitor profile of the Hornbill Festival during 2023–2025.

Year	Total Visitors	Foreign Tourists	Visitors from Other Indian States	Local Visitors (Nagaland)
2023	154,057	2108	37,089	114,860
2024	205,968	2527	56,217	147,224
2025	214,493	2528	56,431	155,534

Source: https://tourism.nagaland.gov.in/ (accessed on 6 July 2026).

**Table 2:** Traditional dishes of different Naga ethnic groups documented at the Hornbill Festival in 2023, 2024 and 2025, Nagaland.

Sl. No.	Traditional Dishes	Ethnic Group	Species	Type of Meat	Ingredients	Cooking Method(s) and Time	Appearance (A) and Consistency (C)
1.	Moudi	Angami	Mithun or beef and pork	Fresh meat	Big chunks of Mithun meat or beef and pork, water, ginger, garlic, red chili powder, salt and blood of Mithun or cattle.	Boiling and simmering, 16 to 20 h for 500 kg meat	A: Dark brown color C: Dry
2.	Galho	Angami	Beef, pork or chicken	Fresh meat (preferred) or smoked	Smoked beef, pork or chicken, rice, water, vegetables, assorted wild leaves, fermented soybeans (axone: only with smoked meat), ground green chilies and red chili powder.	Boiling and simmering, and 60 min	A: Greenish white C: Thin and soupy to thick and creamy
3.	Anishi/Nuoshi	Ao	Beef, pork or eel	Smoked	Smoked beef, pork or eel, fermented Colocasia leaves, water, king chili, green chilies, tomatoes, salt, ginger and garlic.	Boiling, 45 to 60 min	A: Dark brown to black C: Thick curry
4.	Amrusu	Ao	Chicken or pork innards	Fresh meat	Chicken or pork innards, ground rice, water, bamboo shoot juice, green chilies, salt, ginger and garlic.	Boiling and simmering, 45 to 60 min	A: Creamy off-white C: Soupy to thick
5.	Smoked pork with axone	Sumi	Pork	Smoked	Smoked pork, axone (fermented soybeans), water, salt, ginger, garlic and red chili.	Boiling and simmering, ≥1 h	A: Reddish brown C: Thin to thick curry
6.	Benjung Han (Black sesame curry)	Lotha	Smoked and dried pork or beef and fish	Pork or beef: smoked and dried Fish: fermented and dried	Bite-sized smoked and dried pork or beef, fermented dry fish, water, fermented bamboo shoot, ginger, king chilies, and fried and ground black sesame powder.	Boiling and simmering, ≥1 h	A: Blackish C: Thick
7.	Machi Han	Lotha	Fish	Fermented and dried	Fermented dry fish, abundant green chilies, tomatoes, brinjal, water and garlic.	Boiling and simmering, 30 min	A: Greenish C: Thick gravy
8.	Snail with smoked and dried pork	Zeliang	Pork	Smoked and dried	Snails, smoked and dried pork, water, bamboo shoot water, minced ginger, garlic, red chili powder and wild coriander or mint.	Boiling and simmering, 1.5 h	A: Grayish C: Soft
9.	Daona Hon	Kachari	Chicken	Fresh meat	Chicken, water, rice flour, ginger, garlic, chilies and vegetables as per the preferences.	Boiling, 45 min	A: Creamy C: Thin to thick curry
10.	Smoked pork with sesame	Khiamniungan	Pork	Smoked	Smoked pork, sesame seeds, water, rice bean (*Vigna umbellate*), dry chili, salt and Sichuan pepper.	Boiling and simmering, 1 h	A: Brownish C: Thin to thick curry
11.	Mepoh	Kuki	Pork, chicken or beef	Fresh or smoked meat	Sijou leaves (harvested from forest), rice grist, water, changal (a locally crafted liquid soda derived through the filtration of ashes from burnt firewood), salt and chili powder.	Boiling and simmering, 1 h	A: Greenish white C: Thick
12.	Pig trotters in kholar beans	Yimkhiung	Pig trotters	Fresh meat	Pig trotters, kholar or kidney beans (*Phaseolus vulgaris*), water, dried chili, garlic and ginger.	Boiling and simmering, >1.5 h	A: Brownish C: Thin curry
13.	Anphet (Aan-Phat)	Phom	Pork	Fresh meat	Fresh pork, water, soaked rice, ginger–garlic paste, salt, and green chilies.	Steaming: if a bamboo shaft is used, 1 h Simmering: if directly added to water in a pot, 1 h	A: Grayish brown C: Spongy
14.	Smoked pork with perilla	Sangtam	Pork	Smoked	Smoked pork, axone (fermented soybeans), local chili powder, salt, and ground Perilla (*Perilla frutescens*) seeds.	Boiling and simmering, 1 h	A: Brownish C: Thick
15.	Wak kappa or pork kappa	Garo	Pork	Fresh meat	Pork, water, green vegetables, tapioca leaves, ginger, salt, green chilies and kalchi or baking soda.	Boiling and simmering, 1 h	A: Creamy greenish C: Thin
16.	Nasang süru/vin süru	Thikhir	Pork	Smoked	Smoked pork, pork lard, rice bean *(Vigna umbellate*), chopped spring onions, salt and chili powder.	Boiling and simmering, 45 min	A: Creamy C: Semi-dry

## Data Availability

The original contributions presented in this study are included in the article/Supplementary Materials. Further inquiries can be directed to the corresponding author.

## Supplementary Materials

The following supporting information can be downloaded at https://www.mdpi.com/article/10.3390/foods15172991/s1. Figure S1: Illustrates different meat, legume and fish processing methods. A, B and C. Traditional method of hot smoking of meat by hanging and placing it on different structured platforms made out of bamboo; D. Hot smoking of pork skin; E. Traditional axone (fermented soybean) preparation involves wrapping boiled soybeans in banana or *Stachyphrynium placentarium* leaves and fermenting them above a fireplace for a week; F. Hot smoking and drying of meat product under sun; G. Dried lard under shade; H. Sun-dried fish; I. Fermented and sun-dried fish. Figure S2: Illustrates the food serving or packaging materials. A. Areca nut plates; B. Food served on plates made of areca nuts with banana leaves underneath; C. Aluminum cups; D. Banana leaf for serving food; E. Rice served wrapped in a banana leaf; F. *Stachyphrynium placentarium* leaves for wrapping or serving meat products or rice; G. Reusable bamboo plate with *Stachyphrynium placentarium* leaves underneath; H. Food served in Styrofoam cups; I. Laminate pouch for meat products packaging. Table S1: Different cooking techniques of traditional dishes from different Naga ethnic groups documented at the Hornbill Festival in 2023, 2024 and 2025, Nagaland.
