# Supplementary material for: A Survey of Traditional Animal-Origin Foods and Preliminary Assessment of Market Characteristics at the Hornbill Festival in Nagaland, India
Source: Foods. 2026 Aug 26;15(17):2991. doi: 10.3390/foods15172991 (PMC13564595; doi:10.3390/foods15172991)
Supplement: Supplementary file 1 [file foods-15-02991-s001.zip › foods-4454431-supplementary.pdf]

Table S1. Different cooking techniques of traditional dishes from different Naga ethnic groups documented at the Hornbill Festival in 2023, 2024 and 2025, Nagaland.

Sl. No.	Name of the product	Meat used and cooking process	Photo of product
Ethnic group: *Angami*			
1.	Moudi	It is a highly revered cuisine of the Angami tribe and is usually prepared during special occasions by the elders in the community who have years of experience in preparing. The meat of Mithun is preferred the most. To begin, boil water and add local red chilli powder, when water gets boiled the big chunks of meat of Mithun or beef and pork or combination (Mithun/beef and pork) are introduced into large utensils for cooking for hours under firewood and stirred continuously. Blood of Mithun or cattle is also added and stirred continuously until the meat is cooked. The ginger, garlic and salt are added to increase flavour and delicacy, and covered with banana leaf for simmering. After the meat is thoroughly cooked, it is kept in a cool place to dry off before packing in banana leaves.	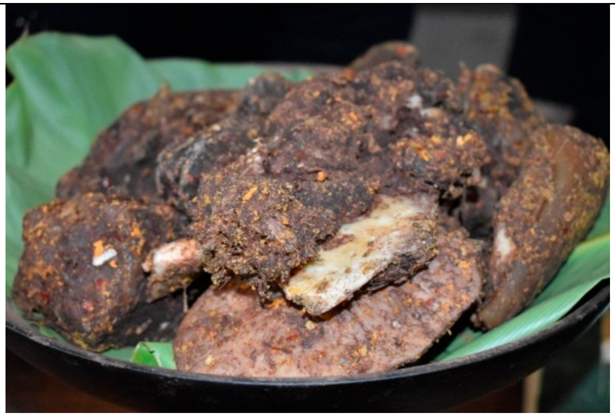
2.	Galho	Galho, a traditional dish originating from the Angami tribe, is a flavorful blend of rice, vegetables, assorted wild leaves, and fresh or smoked beef/pork. However, variations in meat or plant leaves can be incorporated based on individual preferences, leading to diverse preparations among the Angami Nagas. To begin, bring water, fermented soybeans (axone: only if smoked meat is used), and ground green chillies to a boil in a pot. Season with salt to taste. Subsequently, introduce beef or pork to the pot, and boil. Later simmer for an additional 30 minutes or until the meat attains tenderness. Add rinsed rice into the pot, adjust water as needed, and cook over medium-high heat. As the rice nears completion, include chopped greens, allowing the mixture to simmer for 5-7 minutes.	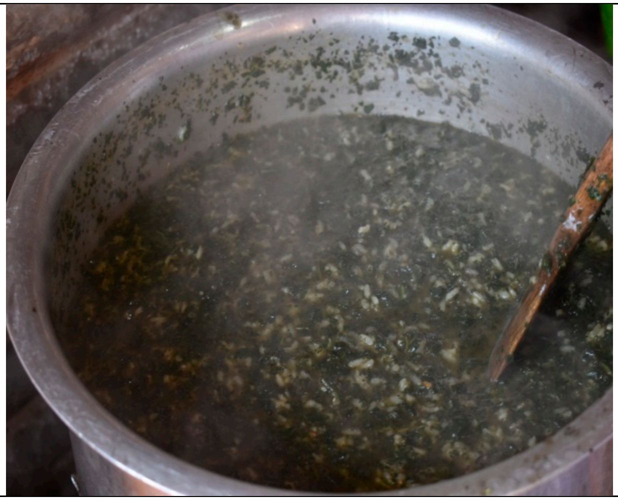
Ethnic group: *Ao*			

3.	Smoked pork Nuoshi / Anishi	Nuoshi is a traditional recipe of the Ao Nagas and is made out of colocasia leaves. The word 'Nuoshi' means fermented yam leaves. 'Nuo' means yam leaves and 'shi' means fermented. The fresh matured colocasia leaves are staked together and kept in a basket and covered until the leaves are fermented. The leaves are crushed until they form a smooth paste. This paste is carefully enveloped in banana leaves and heated on a low flame for several hours until fully cooked. Once cooked, the package is taken off the heat, unwrapped, and shaped into small patties. These patties are then either sun dried or smoked over a fire until they acquire a firm, dark appearance. Nuoshi is best when cooked with smoked pork or smoked beef or smoked eel.<br>To begin, before adding the Nuoshi cakes, warm it in heated charcoal over fire place and slightly pound it to make it little soft. Wash them with water and add them to the pot. To the pot add the smoked pork, king and green chillies, tomatoes, salt and water. Bring them to a boil, cover the pot and cook till the pork and Nuoshi cakes are cooked. Once cooked, take out Nuoshi, chillies, tomatoes and with the help of mortar and pestle pound them and mash them nicely.Add the mashed paste, coarsely grounded ginger and garlic into the pot and boil.	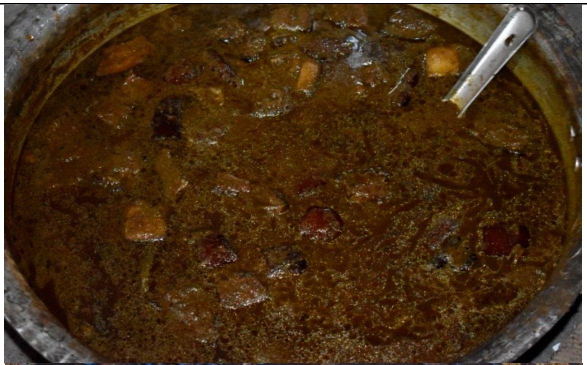 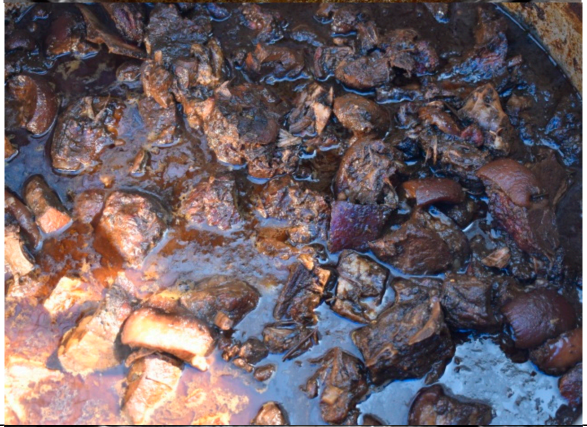
4.	Amrusu	Amrusu, the traditional dish of the Ao Nagas, features a blend of ground rice, chicken or pork innards, and bamboo shoots seasoned with green chillies, ginger, and garlic. To start, soak the rice for 2-3 hours before grinding, and set aside. Combine chicken or pork innards, bamboo shoot juice, and chillies in a pot, add water and simmer on low heat for 20 minutes. Introduce the ground rice to the mixture and continue cooking for an additional 20 minutes. The desired consistency of the final dish can be adjusted according to the consumer preference.	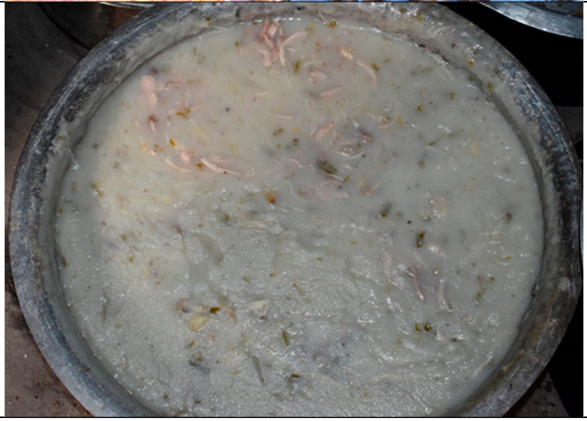
Ethnic group: *Sumi*			

5.	Smoked pork with Axone	<u>Preparation of Axone</u><br>Axone, also known as Akhone, stands out as one of Nagaland highly cherished delicacy, with the sema tribe being renowned for their exceptional axone preparation skills. Initially, soybeans are boiled until they attain a soft consistency, after which the water is drained. The boiled beans are then enveloped in banana or *Stachyphrynium placentarium* leaves, and placed above the fireplace for a week to undergo fermentation. The resulting product can be utilized immediately or stored for later use.<br><br><u>Smoked pork with Axone</u><br>Naga people highly appreciate the delectable combination of smoked pork and Axone. To start the preparation, boil axone, salt, garlic, and red chillies in water for approximately 30 minutes. Subsequently, cleanse the smoked pork with warm water, cut it into small, medium, or large pieces, and cook it with axone until the meat is fully done, and the gravy thickens or reduces to about one-third of its original volume. Remove the boiled red chillies, mash it, and introduce it to the simmering mixture of axone and pork for enhanced color and an appealing appearance.	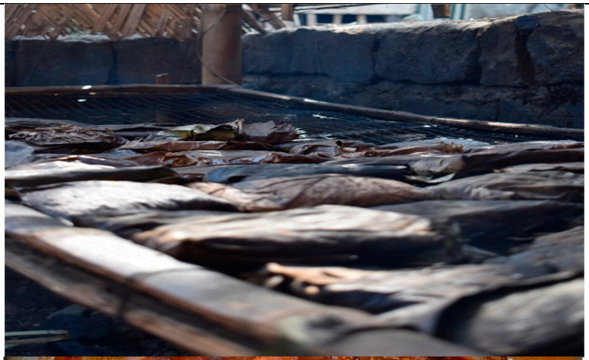<br>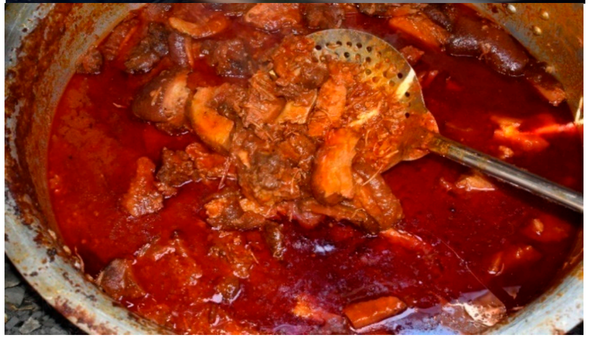
Ethnic group: *Lotha*			
6.	Benjung Han (Black sesame curry)	Benjung Han, a revered dish in the Lotha tribe, is a black sesame curry known for its spiciness. It comes in various forms, with the smoke dried pork or beef benjung curry being the most common. To prepare, start by boiling bite-sized dry pork or beef, fermented bamboo shoot, fermented dry fish, ginger, naga or king chillies and salt in water. Afterward, incorporate fried and ground black sesame powder and simmer for 30 minutes until the water evaporates. Different variations may involve adding or omitting certain ingredients.	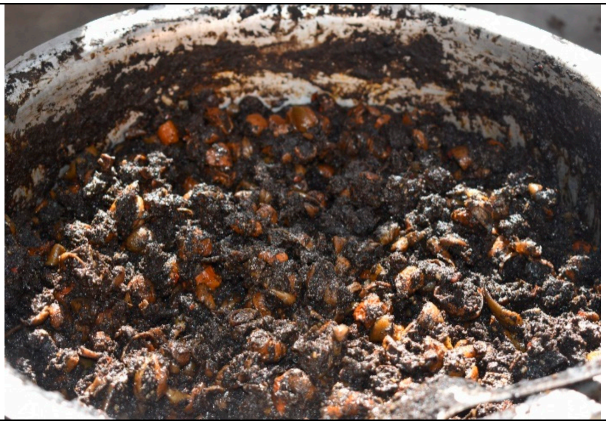
7.	Machi Han	Machi Han, chutney popular within the Lotha tribe, exhibits various versions depending on the clan. There are two primary variations: one includes brinjal, while the other omits it. The key components of this curry comprise abundant green chillies, tomatoes, fermented dry fish, brinjal, water and garlic. All these ingredients are combined and boiled	

		for 20 minutes. The ingredients are taken out and mashed, reintroduced and simmered for 10 minutes. The inclusion of additional vegetables is tailored to the consumer preferences, and the dish is typically served as a side accompaniment. Machi Han is renowned for its synergistic pairing with fresh pork curry, highlighting its catalytic effect on the overall dining experience.	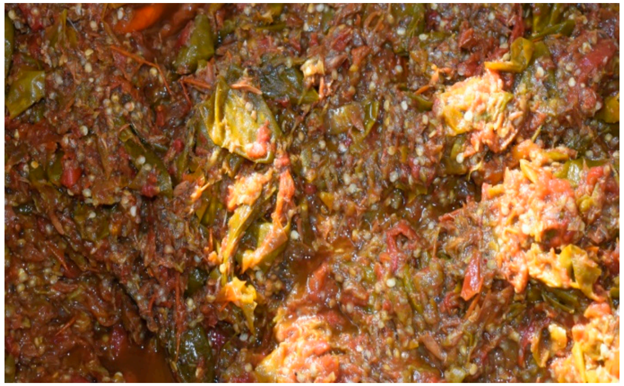
Ethnic group: *Zeliang*			
8.	Snail with Dry pork	Although snail with smoked dry pork is not a traditional food, it is preferred among zeliang tribe. Trim the pointed apex and aperture of the snail. Sprinkle a generous amount of salt and let it soak for a while. Rinse it under running water to eliminate salt and mucus, and if necessary, use a brush to remove stubborn debris. Inspect the shell for any remaining dirt and wash the snail thoroughly. In the cooking process, combine the snails with bamboo shoot water. Add smoked dry pork meat, minced ginger, garlic, red chilli powder, and optionally, wild coriander or mint.	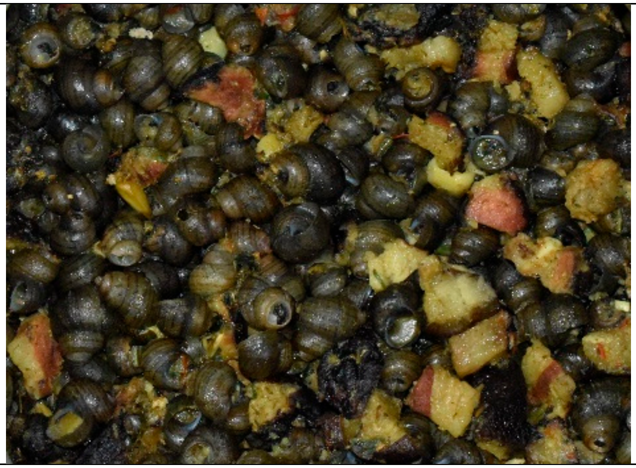
Ethnic group: *Kachari*			
9.	Daona Hon	Daono Hon stands as the traditional ethnic dish of the Dimasa Kachari tribe. Crafted through time-honored methods, this delicacy combines rice powder and chicken. The chicken undergoes a swift cooking process with an assortment of spices and vegetables. Subsequently, warm water is introduced, bringing the mixture to a boiling point. The crucial touch comes with the addition of Hon, the rice powder, imparting enhanced flavor and a distinctive texture to the dish.	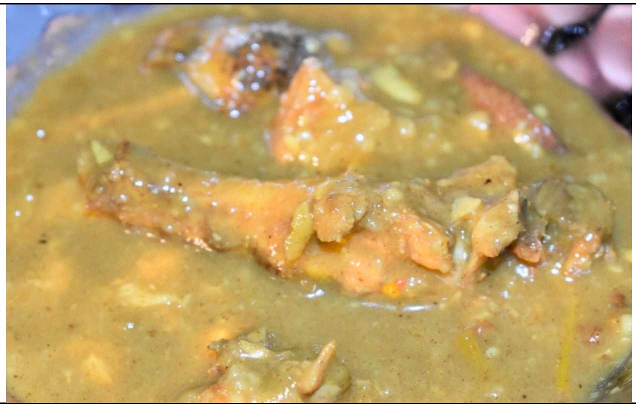

Ethnic group: *Khiamniungan*			
10.	Smoked pork with sesame	Heat water to boiling point and add axone. Allow it to simmer, then incorporate sesame seeds and smoked pork and cook for an hour. Subsequently, introduce rice bean (*Vigna umbellate*), dry chili, salt, and sichuan pepper into the mixture.	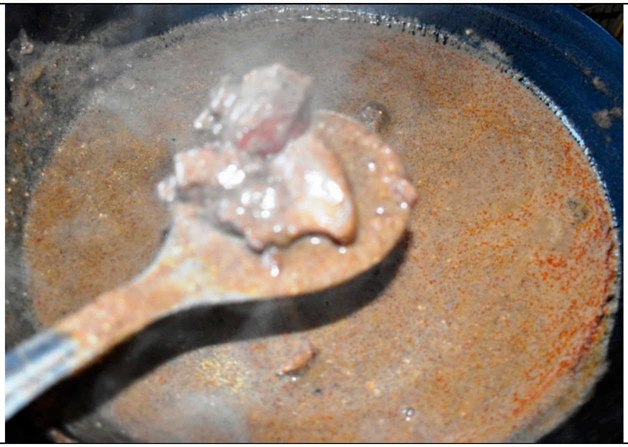
Ethnic group: *Kuki*			
11.	Mepoh	The special dish of the Kuki people is called Sijou Mepoh. It's unique and tasty because it brings together a mix of local ingredients that create amazing flavor. This dish is a wonderful blend of things that come from the local area, making it a special and delicious part of Kuki cuisine. To begin with, clean and wash the sijou leaves (harvested from forest) thoroughly. In a pot combine rice grist, sijou leaves along with cold water. Add changal (a locally crafted liquid soda derived through the filtration of ashes from burnt firewood) when the water is luke warm. Incorporate meat (pork or chicken or beef) in the mixture for flavour. Stir continuously, over medium heat until the rice grist reaches a semi-liquid consistency. Adjust the seasoning to taste and serve. It is usually served with any type of local chutney.	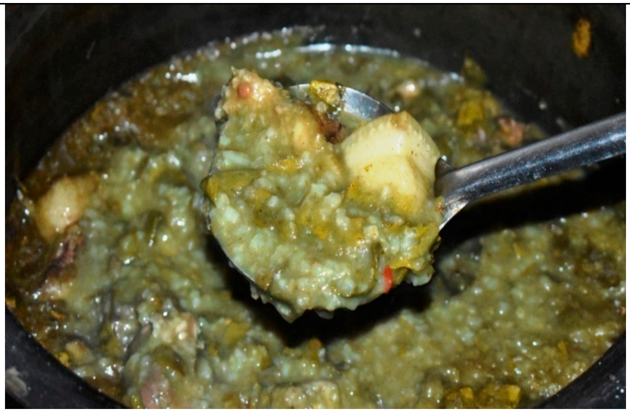
Ethnic group: *Yimkhiung*			

12.	Pig trotters in kholar beans	Trotters consist of the lower part of a pig legs, usually from the ankle downward. Kholar beans, known as Naga kidney beans, are cultivated extensively in the shifting fields of Tuensang and Kiphire districts along the Indo-Myanmar border. To start, simmer the trotters with kholar beans, dried chillies, garlic, and ginger for 45 minutes. Remove the cooked chillies, pound it, then reintroduce it to the dish and continue cooking for an additional hour.	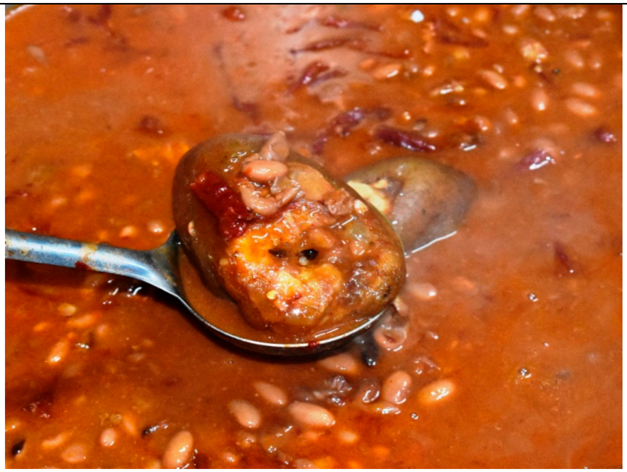 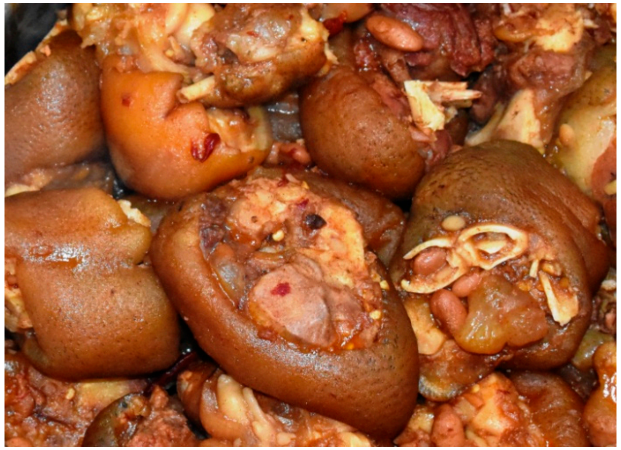
Ethnic group: *Phom*			

13.	Anphet (Aan-Phat)	Aan-phat is a traditional dish commonly prepared during the Monyu festival. To start, immerse ground rice in water for half an hour. Dice the fresh pork into small pieces and combine it in a bowl with the soaked rice, garlic, salt, and green chillies. Some use bamboo shoot water and dried yam leaves according to their preference. Mix thoroughly to create a paste. Ultimately, wrap the mixture in *Stachyphrynium placentarium* leaves or banana leaves and insert it into a bamboo shaft before steaming it in a pot of boiling water for an hour. Alternatively, place the bamboo directly over a fire, rotating it frequently after 10 to 15 minutes or directly add wrapped mixture to water in a pot and simmer for an hour. This dish pairs well with local rice beer and other beverage options.	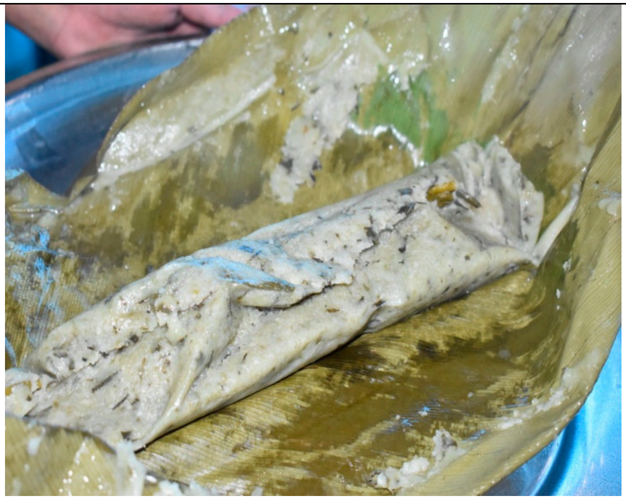
Ethnic group: *Sangtam*			
14.	Perilla with smoked pork	Perilla with smoked pork is not a distinctive dish of the Sangtam tribe; instead, their preference lies in preparing smoked pork with Perilla seeds. The Sangtam tribe traditionally grinds roasted *Perilla frutescens* seeds and sets them aside. To start the cooking process, combine smoked pork, axone, local chilli powder, salt, and the ground Perilla seeds, roasting the mixture until the fat is released. Finally, add water to cover the ingredients and cook for 45 minutes to an hour.	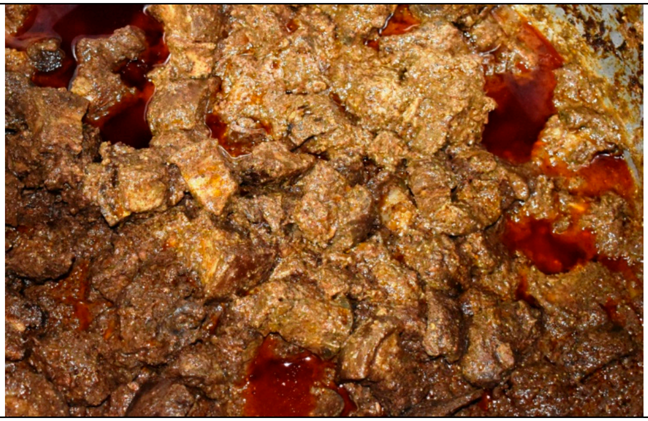
Ethnic group: *Garo*			

15.	Wak kappa or pork kappa	Wak kappa: Wak means "pork" in Garo language. The preparation involves boiling chicken with green vegetables, typically tapioca leaves, ginger, salt and a generous amount of green chillies. A key element that imparts a unique flavor is the inclusion of kalchi or baking soda. This distinctive taste sets it apart. The dish can also be crafted without green vegetables, catering to individual preference. Widely cherished, it is a highly popular dish enjoyed by many. Additionally, the identical recipe can be applied to create Do;o kappa or chicken kapa, offering another delectable culinary delight.	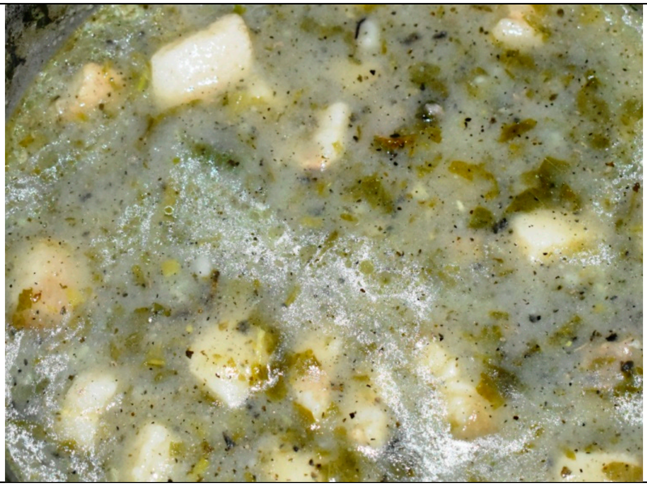
Ethnic group: *Thikhir*			
16.	Nasang süru/ vin süru (Spring onion mixed with naga dal)	Make sure the rice bean (*Vigna umbellate*) is thoroughly cooked. Heat a pan and put a substantial quantity of pork lard. Toss in the beans and incorporate sliced smoked pork into the pan. Include chopped spring onions. Season the mixture with salt and indigenous chilli powder. After thorough mixing and seasoning, dish is prepared to be served.	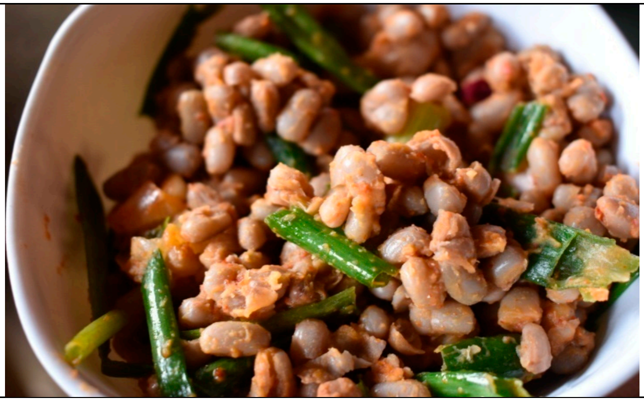

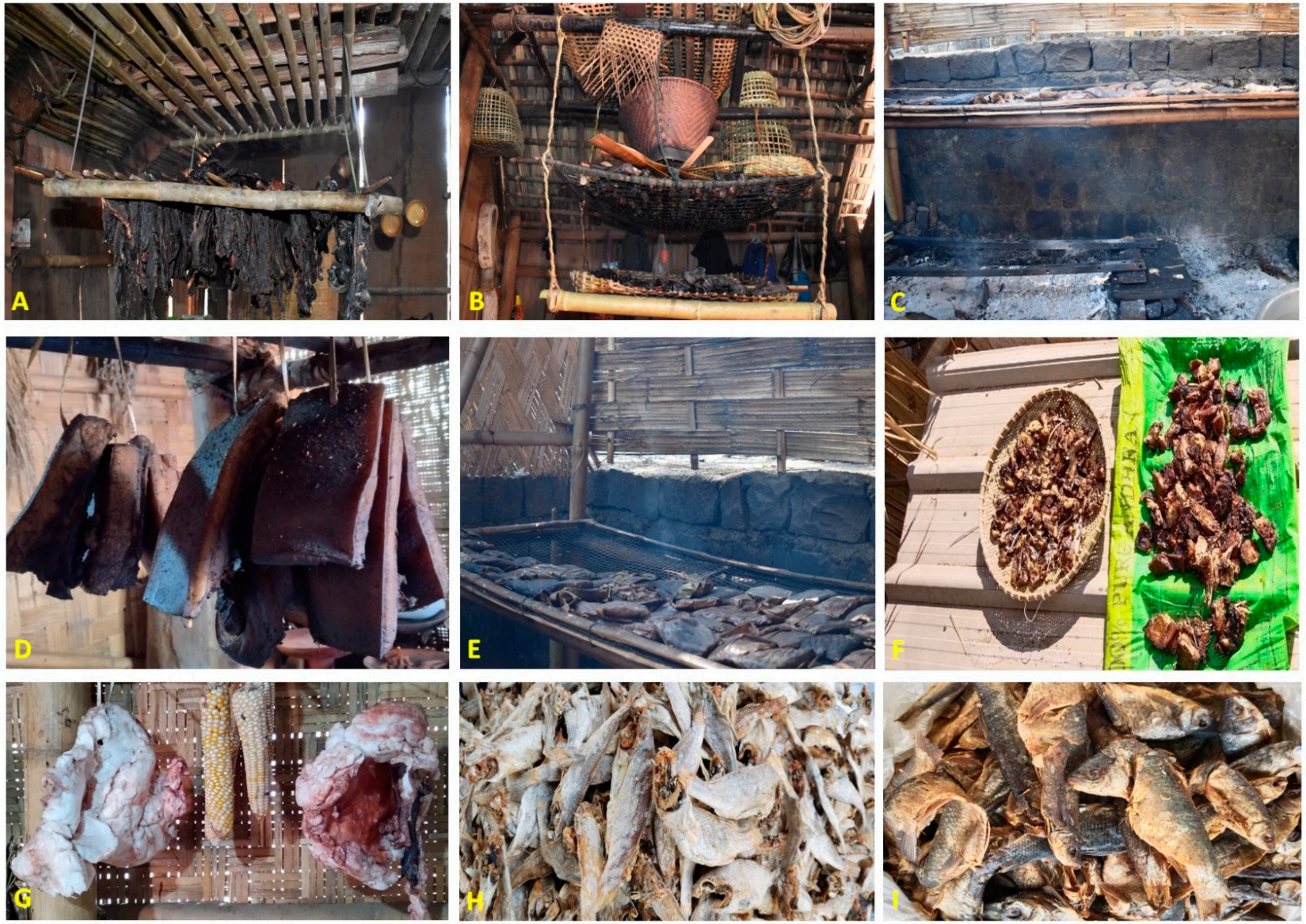

Figure S1: Illustrates different meat, legume and fish processing methods. **A**, **B** and **C**. Traditional method of hot smoking of meat by hanging and placing it on different structured platforms made out of bamboo; **D**. Hot smoking of pork skin; **E**. Traditional axone (fermented soybean) preparation involves wrapping boiled soybeans in banana or *Stachyphrynium placentarium* leaves and fermenting them above a fireplace for a week; **F**. Hot smoking and drying of meat product under sun; **G**. Dried lard under shade; **H**. Sun-dried fish; **I**. Fermented and sun-dried fish.

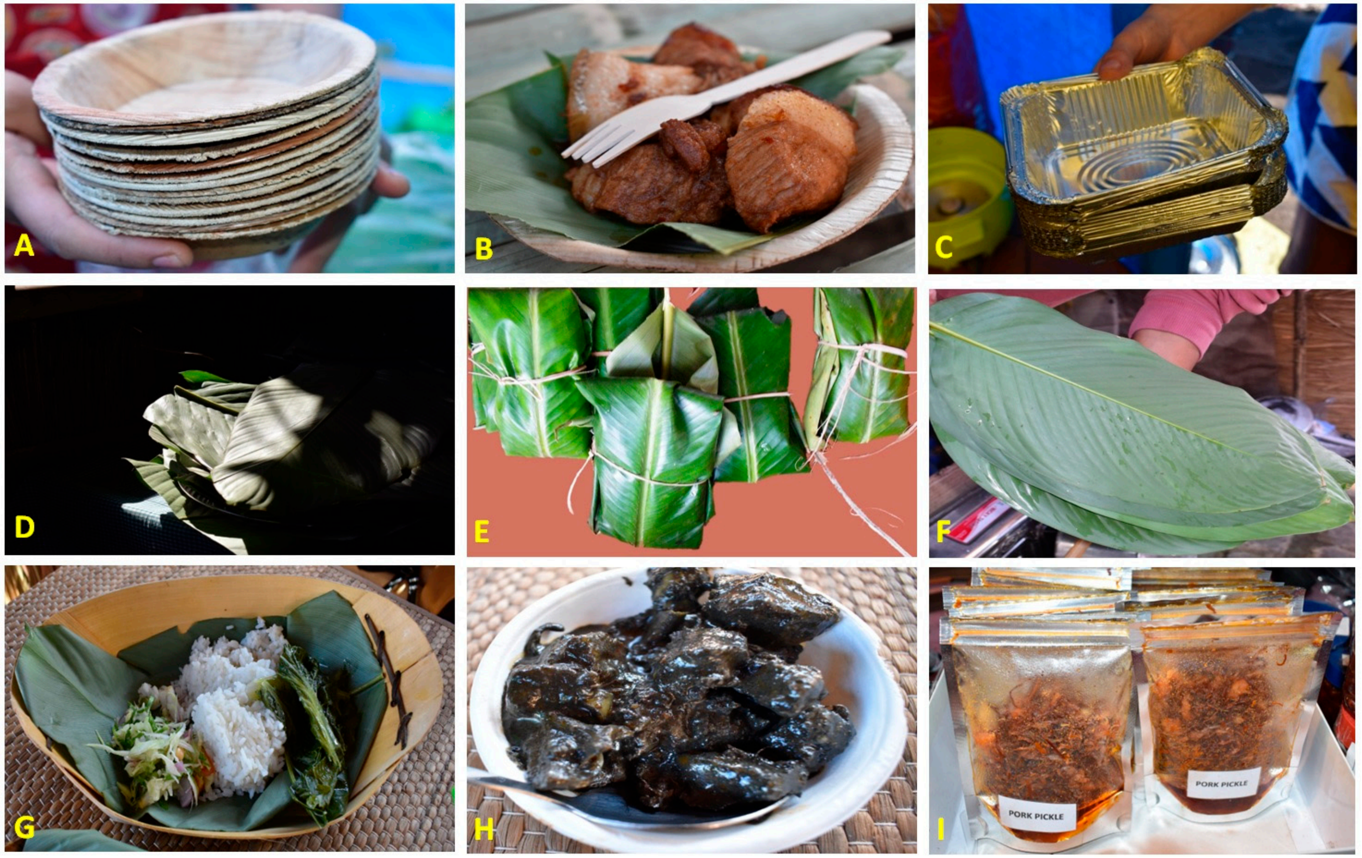

Figure S2: Illustrates the food serving or packaging materials. **A**. Areca nut plates; **B**. Food served on plates made of areca nuts with banana leaves underneath; **C**. Aluminum cups; **D**. Banana leaf for serving food; **E**. Rice served wrapped in a banana leaf; **F**. *Stachyphrynium placentarium* leaves for wrapping or serving meat products or rice; **G**. Reusable bamboo plate with *Stachyphrynium placentarium* leaves underneath; **H**. Food served in Styrofoam cups; **I**. Laminate pouch for meat products packaging.
